# The Cotton WRKY Gene *GhWRKY41* Positively Regulates Salt and Drought Stress Tolerance in Transgenic *Nicotiana benthamiana*

**DOI:** 10.1371/journal.pone.0143022

**Published:** 2015-11-12

**Authors:** Xiaoqian Chu, Chen Wang, Xiaobo Chen, Wenjing Lu, Han Li, Xiuling Wang, Lili Hao, Xingqi Guo

**Affiliations:** State Key Laboratory of Crop Biology, College of Life Sciences, Shandong Agricultural University, Taian, Shandong, China; Texas Tech University, UNITED STATES

## Abstract

WRKY transcription factors constitute a very large family of proteins in plants and participate in modulating plant biological processes, such as growth, development and stress responses. However, the exact roles of WRKY proteins are unclear, particularly in non-model plants. In this study, *Gossypium hirsutum WRKY41* (*GhWRKY41*) was isolated and transformed into *Nicotiana benthamiana*. Our results showed that overexpression of *GhWRKY41* enhanced the drought and salt stress tolerance of transgenic *Nicotiana benthamiana*. The transgenic plants exhibited lower malondialdehyde content and higher antioxidant enzyme activity, and the expression of antioxidant genes was upregulated in transgenic plants exposed to osmotic stress. A β-glucuronidase (GUS) staining assay showed that *GhWRKY41* was highly expressed in the stomata when plants were exposed to osmotic stress, and plants overexpressing *GhWRKY41* exhibited enhanced stomatal closure when they were exposed to osmotic stress. Taken together, our findings demonstrate that *GhWRKY41* may enhance plant tolerance to stress by functioning as a positive regulator of stoma closure and by regulating reactive oxygen species (ROS) scavenging and the expression of antioxidant genes.

## Introduction

During their life span, plants are exposed to various biotic and abiotic stresses. Among these, environmental stresses, particularly drought and salt, which decrease the availability of water to the plant cell, are primary limiting factors for plant growth and development as well as crop yield and quality [1]. To cope with these environmental stresses, plants have evolved intricate acclimatization strategies to avoid or tolerate cellular dehydration.

Plant acclimatization responses usually involve changes to physiological and biochemical parameters. In terms of physiological changes, the stomata play a critical role in the control of water vapor flow [2]. Additionally, plants regulate the expression of genes involved in stress tolerance [3]. In both scenarios, the phytohormone abscisic acid (ABA) plays an important role [4, 5]. It has been well established that water-deficit stress causes significant accumulation of ABA. This increased endogenous ABA content then induces stomatal closure and the expression of various stress-related genes [6, 7]. Numerous transcription factors participate in the ABA response, including HD-ZIP [8], NAC [9], bHLH [10] and WRKY [11]. Among them, WRKY transcription factors, a large family of regulatory proteins, have received much attention in recent decades.

Much research has been performed on WRKY transcription factors since the first WRKY protein was characterized in sweet potato [12]. In *Arabidopsis*, there are 74 family members; rice has more than 100 family members. WRKY genes have also been identified in other species, such as soybean, barley, poplar, *Pine* spp. and *Physcomitrella patens*. All identified WRKY proteins contain either one or two DNA-binding domains consisting of a 60-amino acid region harboring a highly conserved WRKYGQK heptapeptide at its N-terminus with a zinc finger-like motif at its C-terminus. They can be classified on the basis of both the number of WRKY domains and the features of their zinc finger-like motif. Proteins with two WRKY domains belong to group I, whereas most proteins containing one WRKY domain belong to group II. Generally, members of groups I and II have the same zinc finger-like motif, with a pattern consisting of C-X_4-5_-C-X_22-23_-H-X_1_-H. A small subset including members with a C-X_7_-C-X_23_-H-X-C zinc finger-like motif and one WRKY domain is defined as group III [13].

According to previous reports, WRKY proteins are involved in multiple biotic stress responses as well as developmental and physiological processes, including trichome and seed coat development [14], regulation of seed size [15], modulation of leaf development [16] and regulation of leaf senescence [17]. Currently, WRKY proteins are also proposed to be involved in the regulation of plant responses to abiotic stresses. For example, *GsWRKY20* was found to be induced by ABA, salt, drought and cold, and this protein enhanced the drought tolerance of transgenic *Arabidopsis* by regulating ABA signaling. This regulation was achieved due to the ability of GsWRKY20 to promote the expression of a negative regulator and repress the expression of a positive regulator of ABA signaling [18]. Li *et al*. reported that *AtWRKY70* and *AtWRKY54* cooperate to negatively regulate *Arabidopsis* stomatal closure and osmotic tolerance [19]. *TaWKRY2* and *TaWRKY19* were reported to be involved in drought resistance in wheat via direct binding to a downstream gene promoter or via an indirect mechanism [20]. As topics of extensive study, WRKY protein functions have been well examined in model plants, but their roles in crops are not yet clear. Cotton is planted all over the world; its role as a significant source of fiber, oil and biofuel products makes it an important cash crop worldwide [21, 22]. However, current information regarding cotton WRKY proteins is limited. In this report, we identified and characterized a functional WRKY group III gene named *GhWRKY41*. *GhWRKY41* was induced by multiple environmental stresses, and overexpression of *GhWRKY41* in *Nicotiana benthamiana* led to enhanced tolerance to drought and salt. The tissue-specific expression assessed by β-glucuronidase (GUS) staining suggested that *GhWRKY41* was induced in the stoma under salt and drought stresses.

## Materials and Methods

### Plant material, growth conditions and treatments

Cotton (*G*. *hirsutum* L. cv. lumian 22) seeds were placed in wet cloths and germinated. Seedlings were then maintained in hydroponic culture for growth under greenhouse conditions at 25 ± 1°C with a 16-h light/8-h dark cycle (relative humidity of 60–75%). The seedlings were cultured until they were seven days old, at which point they were used for expression analysis. For tissue-specific expression analyses, roots, stems and cotyledons were harvested from the same plant, frozen in liquid nitrogen and stored at -80°C. The resulting uniform seedlings were sprayed or cultured with NaCl (200 mM), 15% polyethylene glycol 6000 (PEG 6000) (w/v), H_2_O_2_ (10 mM), ABA (100 μM), SA (2 mM) and ET released from ethephon (5 mM) or maintained under cold conditions (4°C) or hot conditions (37°C). The treated cotyledons were collected for RNA extraction. *N*. *benthamiana* seeds were surface-sterilized, planted in soil, and maintained under a 16-h light/8-h dark photoperiod at 25°C. Three- or four-leaf-stage *N*. *benthamiana* seedlings were transplanted separately into pots with soil and maintained under greenhouse conditions. The resulting uniform seedlings were used for further study. Each treatment was repeated at least twice.

### *GhWRKY41* cloning, vector construction and genetic transformation

The cDNA and genomic sequence of *GhWRKY41* were isolated as previously described [23]. The primers that were used are shown in S1 Table. The *GhWRKY41* cDNA sequence was cloned into the binary pBI121 vector at the *XbaI* and *SalI* sites; in this vector, the gene was under the control of the *cauliflower mosaic virus* 35S (CaMV35S) promoter. Thereafter, *N*. *benthamiana* was transformed with *Agrobacterium tumefaciens* (strain LBA4404) harboring the recombinant plasmid via the leaf disk method. Transgenic *N*. *benthamiana* seedlings were selected on MS agar medium containing 100 mg/L kanamycin and then transferred into soil in the greenhouse. The T3 progeny of transgenic seedlings were used for functional studies.

### Subcellular localization of GhWRKY41

The open reading frame (ORF) of *GhWRKY41* was inserted into the binary vector pBI121-GFP at the *XbaI* and *XhoI* sites, creating a fusion protein with green fluorescent protein (GFP) fused to the N-terminus of GhWRKY41. The fusion protein was under the control of the CaMV35S promoter. The recombinant plasmid was transformed into *A*. *tumefaciens* (strain GV3101). After overnight cell culture, *A*. *tumefaciens* was harvested by centrifugation and resuspended in infiltration media (for 100 mL: 1 mL of 1 M MES-KOH, pH 5.6, 333 μL of 3 M MgCl_2_, 100 μL of 150 mM acetosyringone). Five-week-old leaves of *N*. *benthamiana* were used for transformation [24]. The fluorescent signal of GhWRKY41-GFP was detected with a confocal microscope (LSM 510 META; Carl Zeiss) after 5 days of transformation. Leaves expressing the 35S-GFP construct were used as a control.

### 3, 3′-Diaminobenzidine (DAB) staining assay and measurement of malondialdehyde (MDA) and reactive oxygen species (ROS)-related enzyme activities

Accumulation of hydrogen peroxide (H_2_O_2_) was detected with DAB staining as previously described. In brief, *N*. *benthamiana* leaves were incubated in DAB solution (1 mg/mL, pH 3.8) for 24 h at 25°C in the dark. After staining, the leaves were soaked in 95% ethanol overnight to remove chlorophyll. MDA levels and catalase (CAT), superoxide dismutase (SOD) and ascorbate peroxidase (APX) activities were spectrophotometrically measured as previously described [25].

### RNA isolation and quantitative real-time PCR (qPCR)

Total RNA was extracted from cotton and *N*. *benthamiana* using the cetyl-trimethyl-ammonium bromide (CTAB) method [26] and TRIzol reagent (Invitrogen, Carlsbad, CA, USA), respectively. First-strand cDNA was synthesized with EasyScript First-Strand cDNA Synthesis SuperMix (Transgen, Beijing, China). qPCR was subsequently performed to analyze the expression patterns of specific genes. *G*. *hirsutum* ubiquitin rRNA, *G*. *hirsutum 18S rRNA* and *N*. *benthamiana β-actin* genes were used separately as the standard controls. The following thermocycler program was used for amplification: 94°C for 5 min, followed by 26–32 cycles of 94°C for 40 s, 50–55°C for 40 s and 72°C for 40 s. Semi-quantitative RT-PCR was used to detect the expression levels of *GhWRKY41* in the T1 progeny of transgenic plants and under *Rhizoctonia solani* treatment. *G*. *hirsutum 18S rRNA* and *N*. *benthamiana β-actin* genes were used separately as the standard controls. The primers used for qPCR are listed in S2 Table.

### β-Glucuronidase (GUS) histochemical staining assay

Transgenic *Arabidopsis* plants harboring a *ProGhWRKY41*::*GUS* construct were generated via the floral dip method. T3 progeny were used to analyze promoter activity and were stained with GUS histochemical staining buffers as previously described [27].

### Determination of stomatal aperture

Expanded leaves were floated in opening buffer (30 mM KCl and 10 mM MES-KOH, pH 6.15) for 2.5 h under a cool white light, and then the appropriate concentrations of ABA, PEG or NaCl solution were added to the opening buffer. After 2 h, the stomatal apertures were measured under a microscope. The aspect ratio was determined using the image processing software ImageJ.

### Transcriptional activation analysis and yeast one-hybrid assay

To investigate its transcriptional activation, the ORF of *GhWRKY41* was amplified using primers containing *NcoI* and *BamHI* sites. The fragment was then introduced into the bait plasmid pGBKT7 (Clontech) and fused to the GAL4 DNA-binding domain. pGBKT7-*GhWRKY41* and the negative control pGBKT7 were transformed into the Y2H Gold yeast strain. The transformed cells were plated onto synthetic defined (SD) medium without tryptophan to identify positive transformants. Then, the positive transformants were grown on SD medium lacking tryptophan, histidine and adenine. The primers used in the transcriptional activation analysis are listed in S1 Table.

To investigate the DNA-binding activity of *GhWRKY41*, a yeast one-hybrid kit was used in accordance with the manufacturer’s protocol (Clontech, USA). The W-box and mW-box fragments were cloned into the pAbAi vector. The background AbA^r^ expression of the strains was tested. The pGADT7 prey vector harboring the *GhWRKY41* ORF (pGAD-GhWRKY41) was transformed into the aforementioned two strains. Yeast cells were also transformed with the empty pGADT7 vector as a control. The transformed yeast cells were first grown in leucine (Leu)- and uracil (Ura)-deficient SD medium (SD/-Leu/-Ura) to ensure the success of the transformation; they were then grown in leucine (Leu)-deficient SD medium (SD/-Leu) containing AbA (500 ng/mL). After culturing at 30°C for 4 days, the plates were examined.

## Results

### Sequence characterization of *GhWRKY41*

Previous studies have shown that group III WRKY family members play important roles in plant defense responses. To clarify the function of this family in cotton, we sought to isolate group III WRKY genes by using homology-based cloning methods. Based on the conserved WRKY domain sequence, a pair of degenerate primers, M1 and M2, was designed to isolate the internal conserved fragment of the cotton WRKY family. The primers 5P1/2, 5P4/4, 3P1/2 and Q1/2 were designed to perform 5′ rapid amplification of cDNA ends (RACE)-PCR, 3′ RACE-PCR and identification of the full-length cDNA sequence.

Full-length *GhWRKY41* cDNA (GenBank accession number: 1693190) contains 1,231 nucleotides, including a 1,068-bp ORF, 36-bp 5′-untranslated region (5′-UTR) and a 127-bp 3′-UTR that was predicted to encode a 355-amino acid protein with a predicted molecular mass of 40 kDa and an isoelectric point of 5.42. A comparison of the protein sequences of GhWRKY41 and other plant WRKY proteins using DNAMAN demonstrated that GhWRKY41 shared high homology with other WRKY proteins; specifically, it was 66.11% homologous to PtWRKY12 (ACV92014.1) (*Populus tomentosa*), 63.09% to BgWRKY (BAG15875.1) (*Bruguiera gymnorrhiza*), 54.85% to GmWRKY41 (XP_003530379.1) (*Glycine max*) and 41.58% to AtWRKY41 (NP_192845.1) (*Arabidopsis thaliana*). The predicted GhWRKY41 protein contains a WRKY domain of approximately 60 amino acids that is composed of a conserved amino acid sequence (WRKYGQK) and a zinc-finger motif (C-C-H-C), indicating that GhWRKY41 belongs to group III of the WRKY family. Moreover, GhWRKY41 contains a nuclear localization signal, KKRK (S1A Fig).

To investigate the evolutionary relationships between WRKY proteins from different species, a phylogenetic analysis was performed based on their amino acid sequences using the software MEGA version 4.1 and the Neighbor-Joining method. The results demonstrated that GhWRKY41 was closely related to group III WRKY family members, including PtWRKY12, BgWRKY and CaWRKY30 (*Capsicum annuum*). These results further suggest that GhWRKY41 belongs to the group III WRKY family (S1B Fig).

### GhWRKY41 is localized to the nucleus and demonstrates transcriptional activation and DNA binding activity

**Fig 1:**
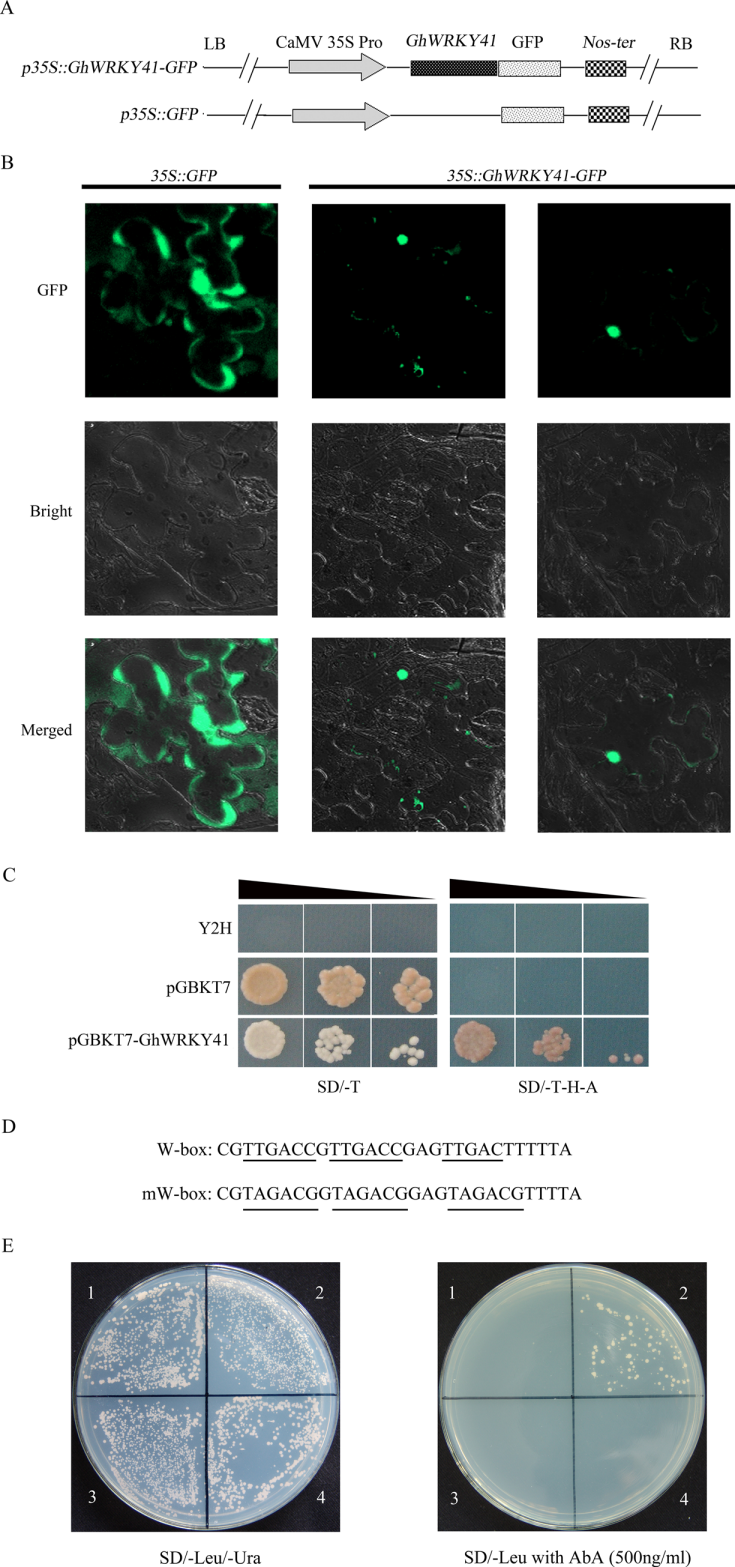
Characterization of GhWRKY41 as a transcriptional regulator. (A) Schematic diagrams of the p35S::GhWRKY41-GFP fusion construct and the control p35S::*GFP* construct. (B) Transient expression of the *p35S*::*GhWRKY41-GFP* and *p35S*::*GFP* constructs in *N*. *benthamiana*. Green fluorescence was observed using a confocal microscope five days after transformation. (C) GhWRKY41 demonstrates transactivation activity. The full-length ORF of *GhWRKY41* was subcloned into pGBKT7, and transformed yeast was selected on both SD-Trp and SD-Trp-His-Ade media. Positive transformants were further identified by spotting serial yeast dilutions (1/1, 1/10 and 1/100). The triangle indicates the dilutions from 1 to 100. (D) Sequences of three tandem W-boxes (TGAC) and mW-boxes. (E) Yeast one-hybrid assays. (1) pAbAi-Wbox + pGADT7. (2) pAbAi-Wbox + pGAD-GhWRKY41. (3) pAbAi-mWbox + pGADT7. (4) pAbAi-mWbox + pGAD-GhWRKY41.

Results from the online protein subcellular location predictors Plant-mPloc (http://www.csbio.sjtu.edu.cn/bioinf/plant-multi/) and PSORT (http://psort.hgc.jp/form.html) suggested that GhWRKY41 was located in the nucleus. Additionally, a putative nuclear localization signal (KKRK) was identified in GhWRKY41. To confirm the nuclear localization of GhWRKY41, we constructed a vector that expressed a GhWRKY41-GFP fusion protein under the control of the CaMV35S promoter. A vector expressing *p35S*::*GFP* was also constructed to serve as the control. Plasmid vectors were individually introduced into *N*. *benthamiana* leaves, and fluorescence was observed with a confocal laser scanning microscope. The results showed that the GhWRKY41-GFP fusion protein emitted green fluorescence predominately in the nuclei, whereas GFP alone was found in multiple subcellular compartments, including the cytoplasm and nucleus. These results suggest that GhWRKY41 is a nuclear-localized protein (Fig 1A and 1B).

To characterize the transcriptional activation activity of the GhWRKY41 protein, the plasmids pGBKT7-*GhWRKY41* and pGBKT7 (negative control) were transformed into the yeast strain Y2H containing the upstream activating sequence (Clontech, UAS), which is specifically bound by the GAL4 binding domain. All transformants grew well on selective medium without tryptophan (SD-Trp). Moreover, transformants harboring pGBKT7-*GhWRKY41* grew on selective medium lacking tryptophan, histidine and adenine (SD-Trp-His-Ade), but transformants harboring pGBKT7 did not grow on this media. These results indicated that GhWRKY41 could transactivate the expression of both HIS3 and ADE2 reporter genes in yeast, suggesting that GhWRKY41 may be a transcriptional activator (Fig 1C).

A yeast one-hybrid assay was performed to test the interaction between GhWRKY41 and the W-box *cis*-element. Yeast strains harboring the W-box and mW-box constructs did not grow in SD/-Ura containing AbA (500 ng/mL). Therefore, 500 ng/mL AbA can suppress the basal expression of the pAbAi vector. pGAD-GhWRKY41 was transformed into the two yeast strains. The empty vector (pGADT7) was also introduced into the two yeast strains to serve as a control. As shown in Fig 1E, all of the yeast cells grew in SD/-Leu/-Ura. However, only the yeast cells carrying the W-box construct and pGAD-GhWRKY41 grew in SD/-Leu containing AbA (500 ng/mL). These data indicated that GhWRKY41 specifically binds to the W-box in yeast.

### Constitutive expression of *GhWRKY41* in whole plants

A 1024-bp *GhWRKY41* promoter fragment was isolated using the hiTAIL-PCR method and analyzed using PLACE and PlantCARE online software. Many abiotic stress-, biotic stress- and development-related *cis-*acting elements were identified (S3 Table).

To determine the expression pattern of *GhWRKY41*, the isolated promoter sequence of *GhWRKY41* was cloned into the pBI121 vector to drive the expression of the GUS protein.

**Fig 2:**
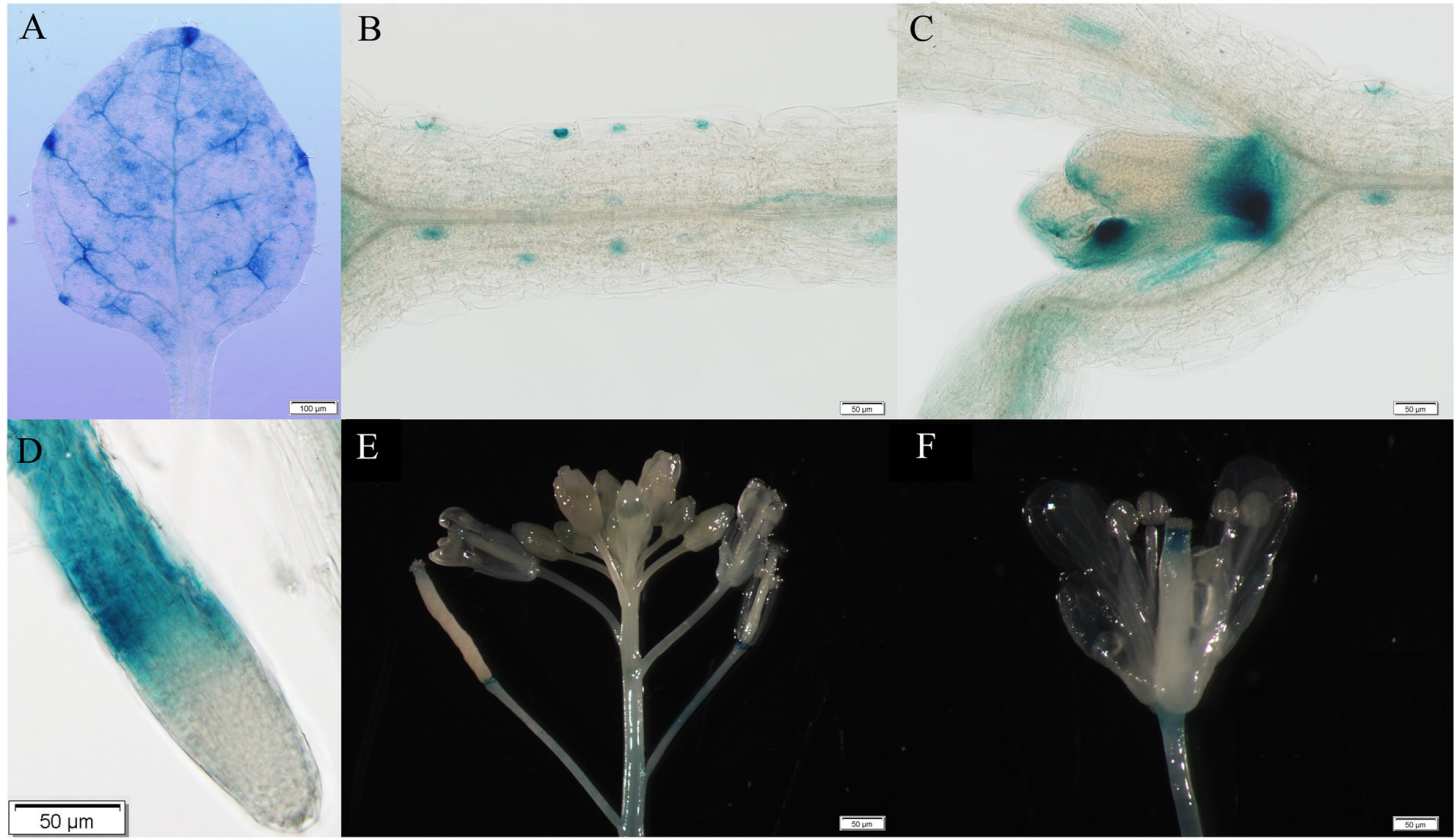
Spatiotemporal expression patterns of *ProGhWRKY41*::*GUS* in transgenic *Arabidopsis*. (A) Leaf, (B) stem, (C) bottom of bud, (D) root, (E) flower and (F) young silique flower. Bars are presented in the lower left corner.

The staining assay showed that *GhWRKY41* was expressed in most plant organs, including leaves, stems and roots (Fig 2A–2D). During the reproductive growth stage, staining was observed in the lower portion of the stigma, but no GUS signal was observed in pods (Fig 2E and 2F). Interestingly, a strong GUS signal was observed at the nodes, indicating probable roles for *GhWRKY41* in plant development (Fig 2C).

### Expression of *GhWRKY41* is regulated by multiple abiotic stresses and signaling factors

**Fig 3:**
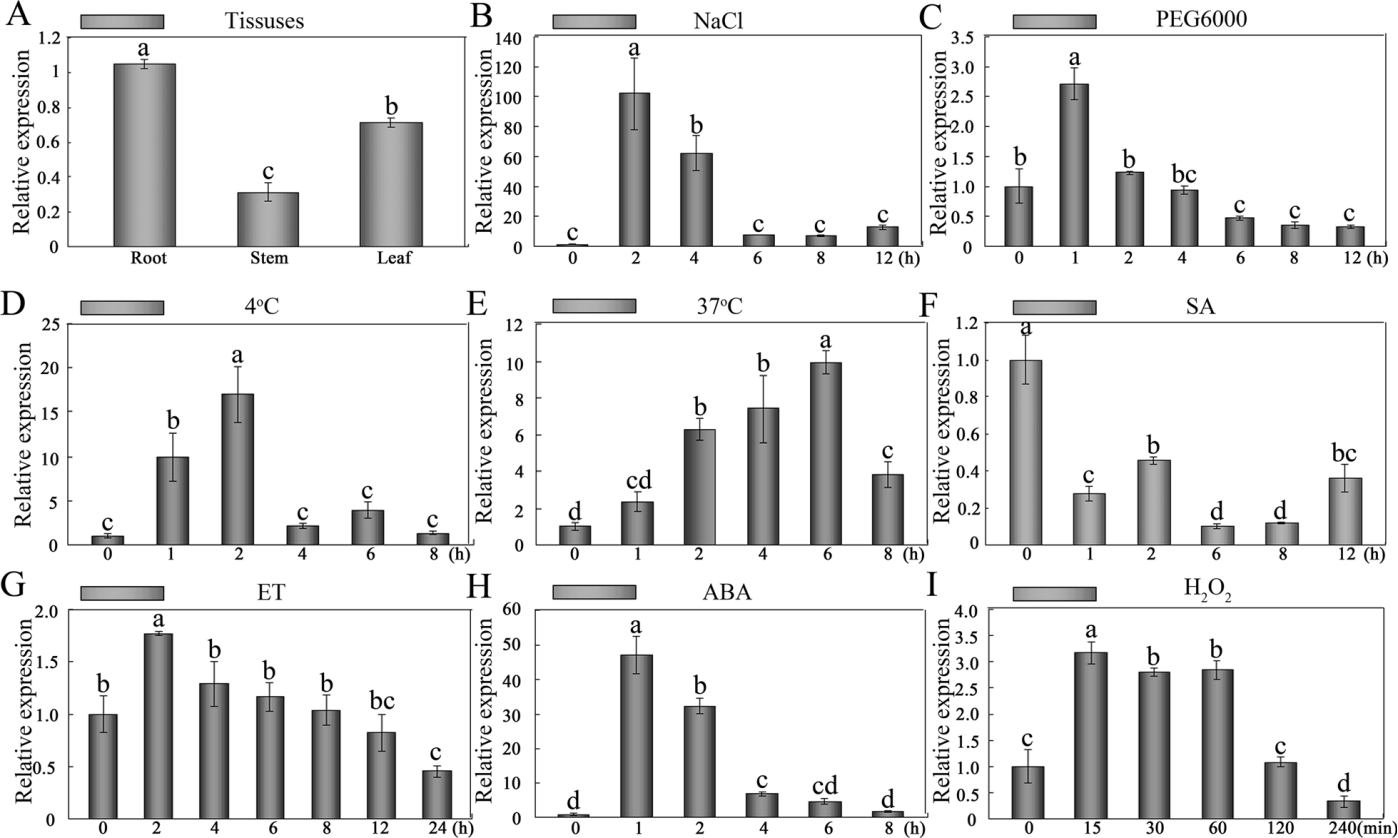
Relative expression of *GhWRKY41* in different tissues and in response to different stress factors. Quantification of *GhWRKY41* band intensity based on the expression of *GhUbiquitin rRNA* genes using Quantity One software. The roots, stems and cotyledons of 7-day-old cotton seedlings were used to assess the tissue-specific expression of *GhWRKY41* (A). The seedlings were treated with 200 mM NaCl (B), with 15% PEG (C), at 4°C (D), at 37°C (E), with 2 mM salicylic acid (SA) (F), with 5 mM ethylene (ET) released from ethephon (G), with 10 μM abscisic acid (ABA) (H) or with 10 mM H_2_O_2_ (I).

qPCR was performed to investigate the expression pattern of *GhWRKY41*. The expression of *GhWRKY41* was higher in the leaf and root compared with the stem (Fig 3A). To further examine *GhWRKY41*, its transcript levels under treatment with various abiotic stresses, hormones and molecular signaling factors were analyzed by qPCR. As shown in Fig 3B–3E, abiotic stresses, including salt, drought, cold (4°C) and heat (37°C), markedly induced the expression of *GhWRKY41*. However, the induction patterns were different. NaCl treatment caused rapid, transient and powerful induction of *GhWRKY41*. Induction by PEG was also transient and rapid. mRNA began to accumulate immediately upon heat treatment and reached a maximum level at 6 h. Under cold treatment, transcript levels were increased at 1 h after the initiation of treatment and reached a maximum at 2 h.

Plant hormone and signaling factors are involved in multiple processes and regulate various signaling pathways. We also characterized the gene expression of *GhWRKY41* under different hormone treatments. As shown in Fig 3F–3I, under ethylene (ET) treatment, the expression level of *GhWRKY41* reached a peak at 2 h and then decreased back to its original level. ABA and H_2_O_2_ treatment also induced gene expression. However, salicylic acid (SA) treatment significantly repressed gene expression.

### Overexpression of *GhWRKY41* enhances drought tolerance of transgenic tobacco

To further investigate the function of *GhWRKY41* in plants, we constructed transgenic *N*. *benthamiana* plants that overexpressed *GhWRKY41*. The transgenic seedlings were characterized by PCR. Seven independent T_1_ lines were selected on kanamycin, and the expression of *GhWRKY41* was assessed. Based on the results, three representative lines (20, 24 and 28) that exhibited different expression levels were selected for further research. Lines 20, 24 and 28 were renamed OE1, OE2 and OE3, respectively, and T_3_ progeny of these lines were used for functional analysis (S2 Fig).

**Fig 4:**
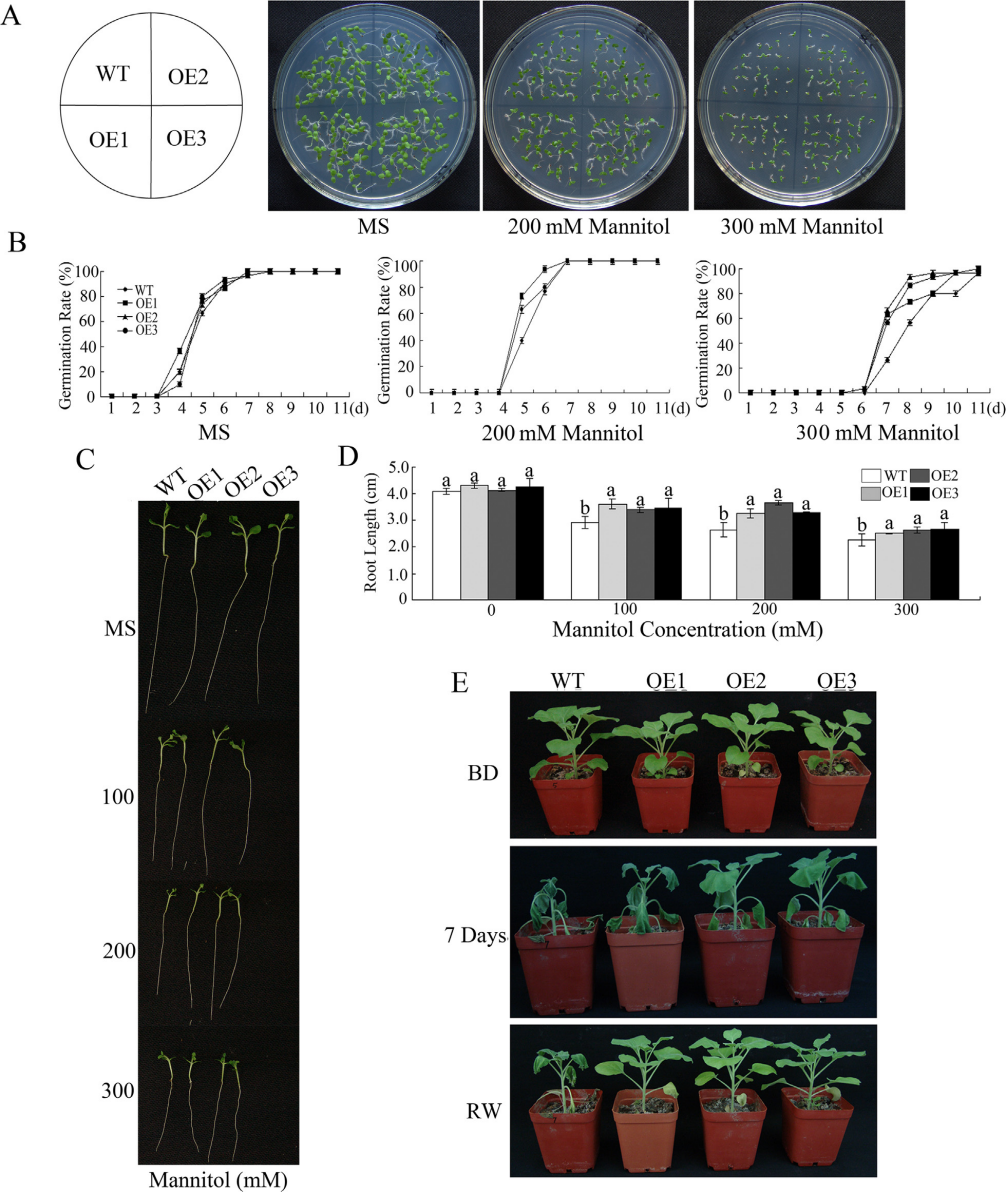
Drought tolerance test comparing wild-type and *GhWRKY41*-overexpressing *N*. *benthamiana* plants. (A, B) Seed germination assay. (C) Post-germination seedling development of the WT and OE lines on MS supplemented with different concentrations of mannitol. (D) Primary root lengths of the seedlings 20 days after germination in the presence of different concentrations of mannitol. (E) Photograph of representative 8-week-old WT and OE plants grown in soil under drought conditions for 7 days and then watered for 3 days to allow them to recover.

To evaluate the influence of *GhWRKY41* on plant tolerance to drought, mannitol was used to mimic a water deficit. Transgenic tobacco seeds were surface-sterilized and germinated on MS agar medium containing 0, 200 or 300 mM mannitol. As shown in Fig 4A, there was no visible difference between the wild-type (WT) and transgenic (OE) lines on medium without mannitol. However, when the concentration of mannitol reached 200 mM, the transgenic lines began to exhibit earlier germination compare with the WT line, although all plants eventually showed a similar germination ratio (Fig 4B). Next, WT and OE seeds were germinated on MS agar medium and transferred to medium that contained mannitol to assay their growth after germination. The results showed that the root lengths of the WT and OE lines were similar on MS medium, but OE plants exhibited longer roots than WT plants on medium with mannitol (Fig 4C and 4D).

To confirm *GhWRKY41* function during vegetative growth, two-month-old plants grown in soil were deprived of water for drought treatment. As shown in Fig 4E, compared with WT plants, in which whole plants exhibited a severe wilt phenotype, transgenic plants suffered a slight wilt. Furthermore, transgenic plants recovered more rapidly than WT plants when they were rewatered 3 days following drought treatment.

**Fig 5:**
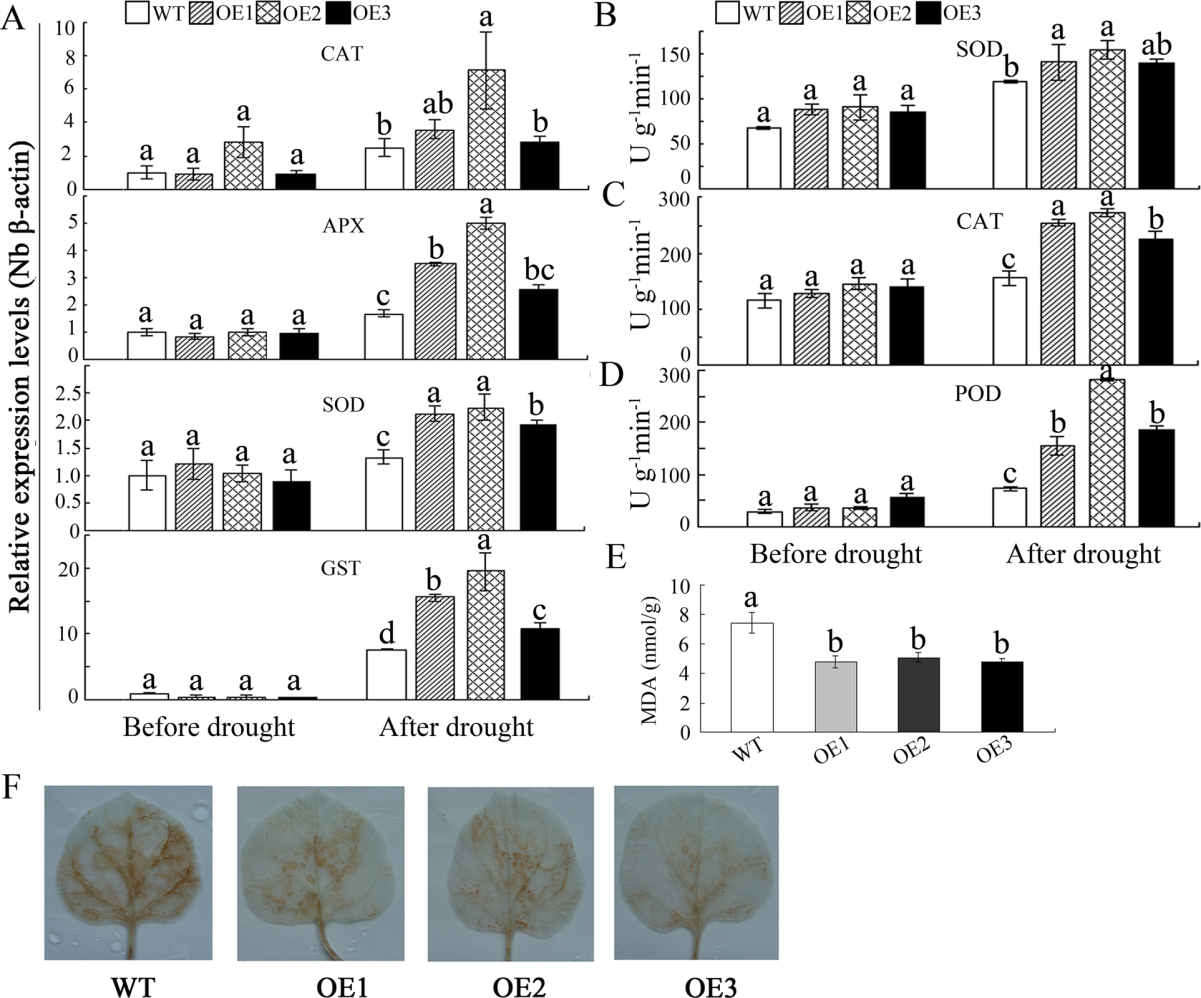
Drought tolerance of WT and *GhWRKY41*-overexpressing *N*. *benthamiana* plants in the vegetative stage. (A) Expression levels of antioxidant enzyme genes. (B-D) Analysis of antioxidant enzyme activity. (E) Drought-induced MDA accumulation in the WT and OE lines. (F) Drought-induced H_2_O_2_ accumulation detected via DAB staining.

To investigate whether the tolerance of transgenic plants was related to oxidative stresses, MDA and H_2_O_2_ contents were analyzed. Under normal conditions, there was no significant difference between the WT and OE lines (data not shown). After treatment, there were marked increases in MDA and H_2_O_2_ in both WT and OE plants; furthermore, H_2_O_2_ and MDA accumulated to much lower levels in the transgenic lines than in the WT line in response to drought stress (Fig 5E and 5F).

To investigate the possible underlying cause of the decreased H_2_O_2_ accumulation in the transgenic lines in response to drought stress, the activities of major antioxidant enzymes were measured. Upon exposure to drought stress, there were marked increases in the activities of SOD, CAT and peroxidase (POD) in both the WT and transgenic lines. However, these increases were significantly greater in the transgenic lines than in the WT lines in response to drought stress (Fig 5B–5D).

To reveal the molecular mechanisms underlying enhanced drought stress tolerance in the transgenic lines, the expression of several antioxidant enzyme genes was assessed by qPCR. Under normal conditions, there was no significant difference in the expression of these genes. Under drought treatment, the expression levels of genes that encoded ROS-scavenging enzymes, such as SOD, CAT, APX and glutathione S-transferase (GST), were significantly increased in the transgenic lines compared to the WT lines (Fig 5A).

### Overexpression of *GhWRKY41* enhances the salt tolerance of transgenic tobacco

Osmotic stress can be caused by several environmental cues, such as drought, high salinity and low temperature. To elucidate whether the activation of *WRKY41* led to drought tolerance alone or whether tolerance to other abiotic factors could be achieved, WT and transgenic plants were exposed to high-salt stress.

**Fig 6:**
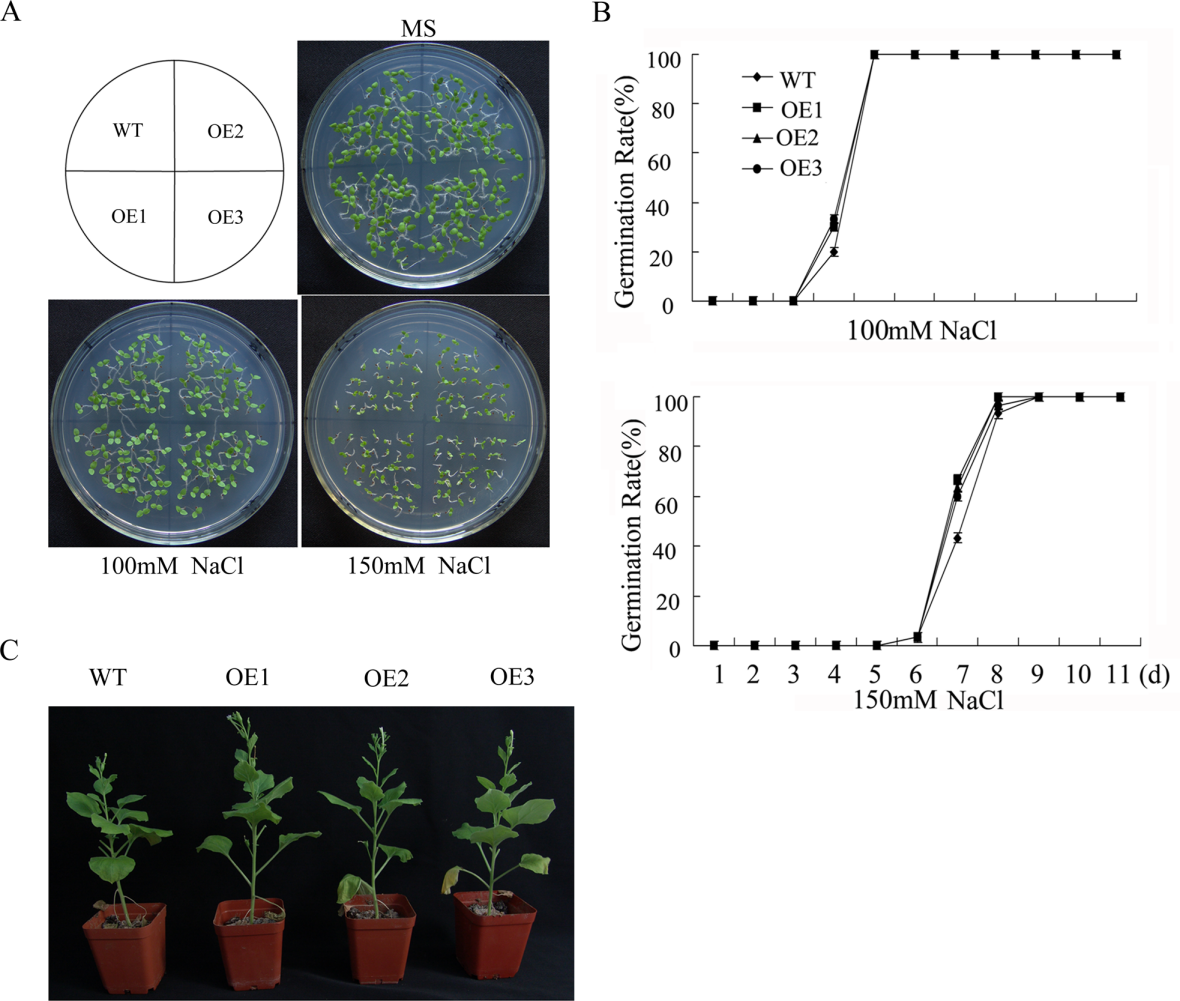
Salt tolerance of WT and *GhWRKY41*-overexpressing *N*. *benthamiana* plants. (A, B) Seed germination assay. (C) Photograph of representative 8-week-old WT and OE plants watered with 200 mM NaCl for 1 month.

Transgenic tobacco seeds were surface-sterilized and germinated on MS agar medium containing 0, 150 or 200 mM NaCl. As shown in Fig 6A and 6B, there was no visible difference between the WT and transgenic lines on medium without NaCl. However, when the concentration of NaCl reached 150 mM, the transgenic lines exhibited earlier germination than the WT lines, although all plants eventually showed a similar germination ratio. Two-month-old plants watered with a 200 mM NaCl solution for 1 month were photographed. As shown in Fig 6C, WT plants were shorter in height and showed severe wilt.

**Fig 7:**
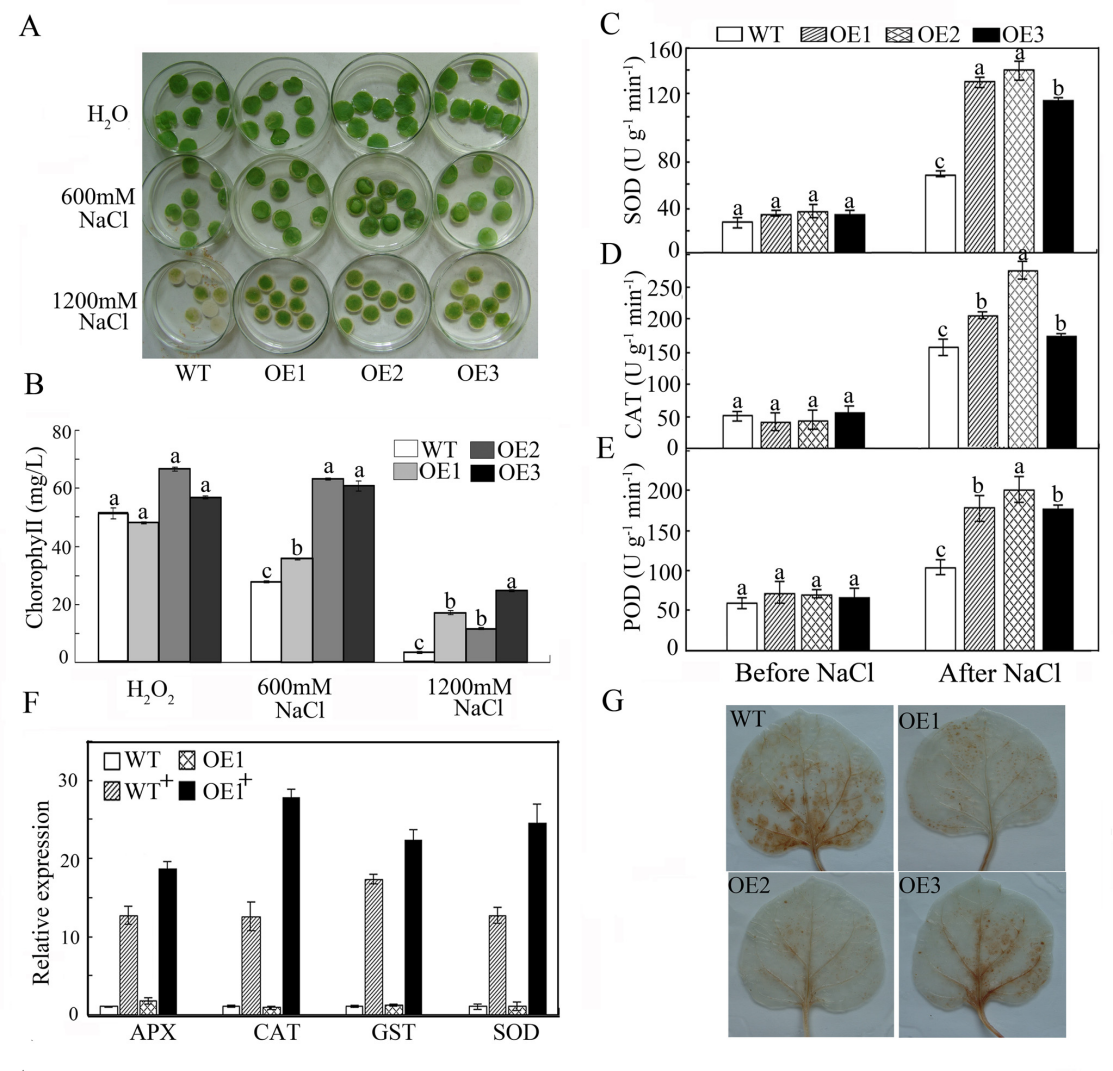
Salt tolerance of WT and *GhWRKY41*-overexpressing *N*. *benthamiana* plants in the vegetative stage. (A) Leaf discs from WT and OE plants were incubated with NaCl at different concentrations (0, 600 or 1,200 mM) under greenhouse conditions. (B) Relative chlorophyll content was determined in the leaf discs of WT and OE plants following NaCl treatments. Disks floated in water were used as controls. The presented data are the means ± SE of three independent experiments (n = 6). (C-E) Analysis of stress-related enzyme activity. (F) Expression levels of stress-related genes. WT^+^ indicates the WT lines after NaCl treatment. OE1^+^ indicates the OE1 lines after NaCl treatment. (G) Salt-induced H_2_O_2_ accumulation detected via DAB staining.

A leaf disk assay was performed to further study the function of *GhWRKY41* at the vegetative stage. Leaf disks detached from two-month-old transgenic and WT plants were suspended in NaCl solutions ranging from 0 mM to 1,200 mM. Leaf disks from both transgenic and WT plants showed no significant changes in water with no added NaCl, although in the NaCl solutions, they all showed signs of bleaching. NaCl treatment caused severe damage in leaf disks from WT plants. This result was further confirmed by measuring leaf disk chlorophyll content before and after NaCl treatment (Fig 7A and 7B).

To determine whether enhanced salt tolerance was related to oxidative stress, we measured the activities of major antioxidant enzymes. After salt treatment, there were marked increases in the activities of POD, CAT and SOD in both the WT and transgenic lines. However, these increases were significantly greater in the transgenic lines than in the WT lines in response to salt stress (Fig 7C–7E). The expression of several abiotic stress-response genes was assessed by qPCR, and the results were consistent with those of the enzyme activity assay (Fig 7F). To intuitively understand the oxidation states of the plants, leaves detached from transgenic and WT plants were stained with DAB. As shown in Fig 7G, WT plants stained darker than transgenic plants under salt stress.

### *GhWRKY41* helps plants to cope with drought and salt stresses by enhancing stomata closure

**Fig 8:**
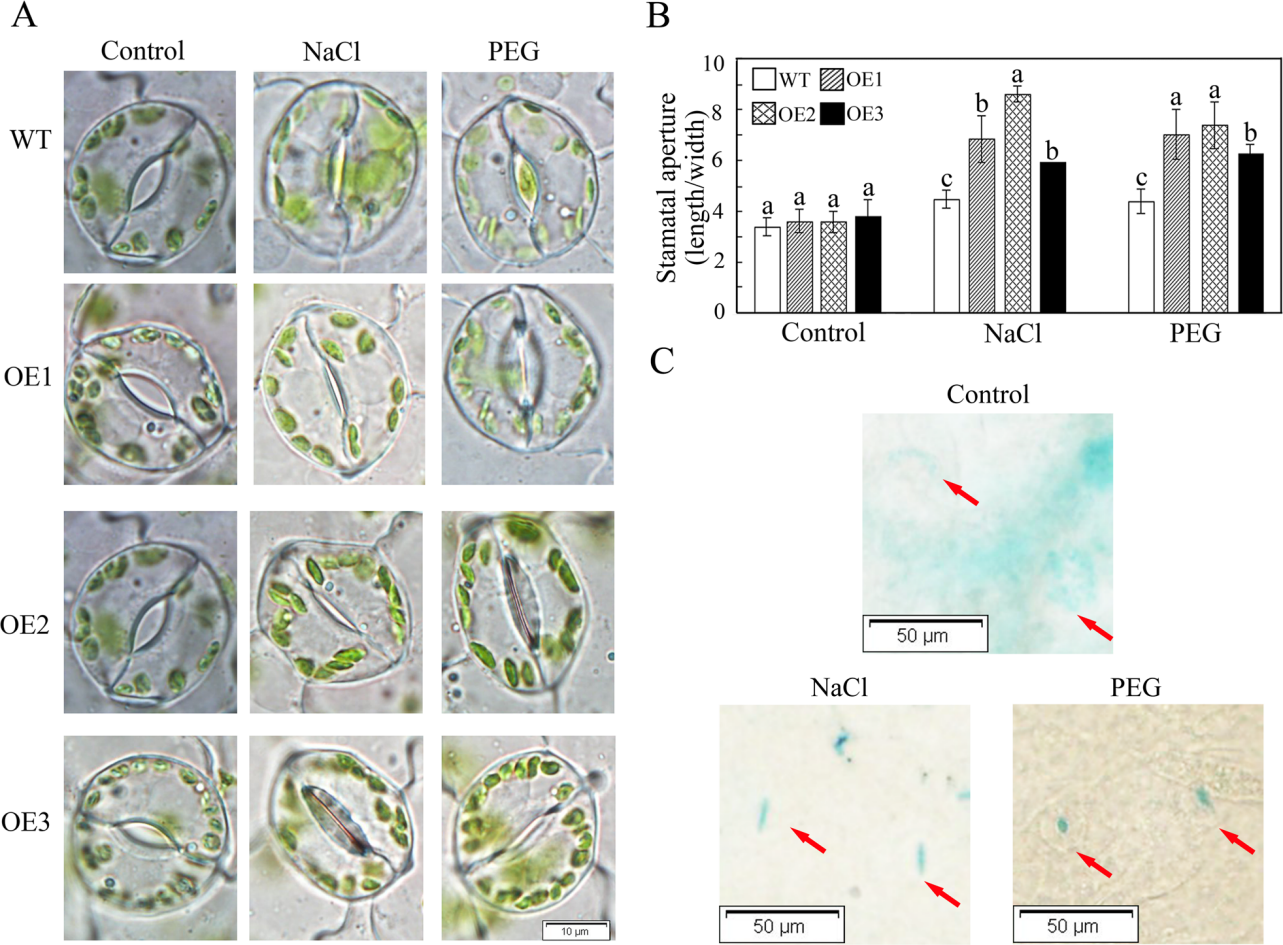
*GhWRKY41* regulates stomatal movement. (A) Comparison of stomatal aperture in response to salt and drought. (B) Stomatal aperture data were calculated from 50 stomata from the leaves of three different plants. Values are the mean ± SD. (C) GUS staining of leaves from transgenic *Arabidopsis* exposed to salt and drought treatments.

In response to drought or osmotic stress, plants are able to control their water content and reduce water loss. Thus, we explored the potential association of water balance regulation with the observed tolerance phenotype. To achieve this goal, we first compared the number of stomata per unit leaf area between WT and transgenic plants, but no significant differences were detected (data not shown). Then, we measured the stomatal aperture of the WT and transgenic lines under control and stress treatments. The transgenic lines exhibited a marked reduction in stomatal aperture (Fig 8A and 8B). And the stomatal conductance was tested by a leaf disk desiccation assay. The result showed that the stomatal conductance was significantly reduced in transgenic plants (S3 Fig). Moreover, in *Arabidopsis* transformed with *proGhWRKY41*::*GUS* and treated with salt and PEG, a GUS staining assay suggested that *GhWRKY41* was highly expressed in stomata (Fig 8C). These results may indicate that *GhWRKY41* enhances drought and salt stresses by regulating stomatal movement.

### *GhWRKY41* enhances osmotic tolerance in an ABA-dependent manner

**Fig 9:**
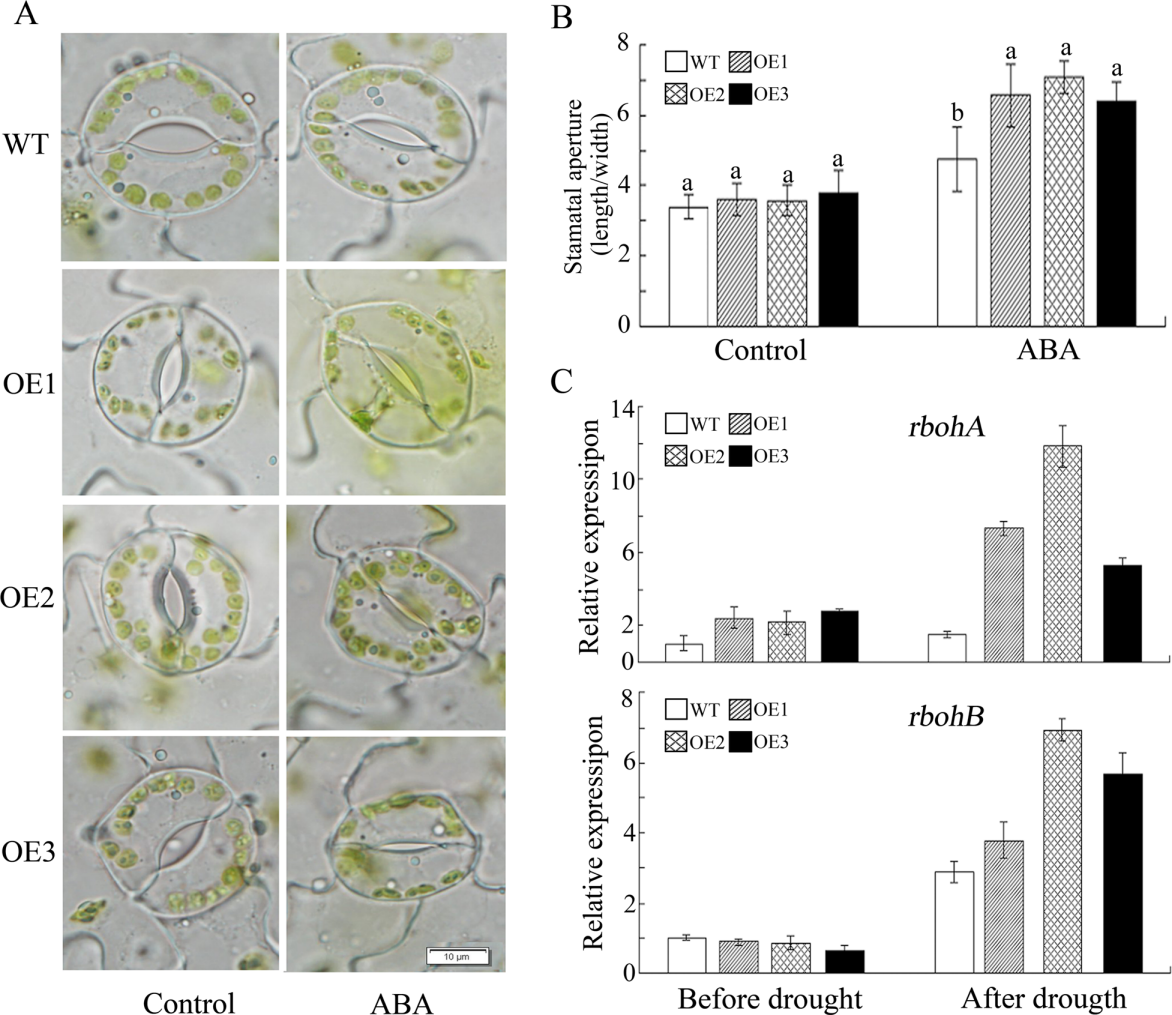
*GhWRKY41* regulates stomatal movement in an ABA-dependent manner. (A, B) Comparison of stomatal aperture in response to ABA treatment. (C) Expression pattern of *rbohA* and *rbohB* genes in WT and OE plants following drought stress. Data were calculated from 50 stomata from the leaves of three different plants. Values are the mean ± SD.

**Fig 10:**
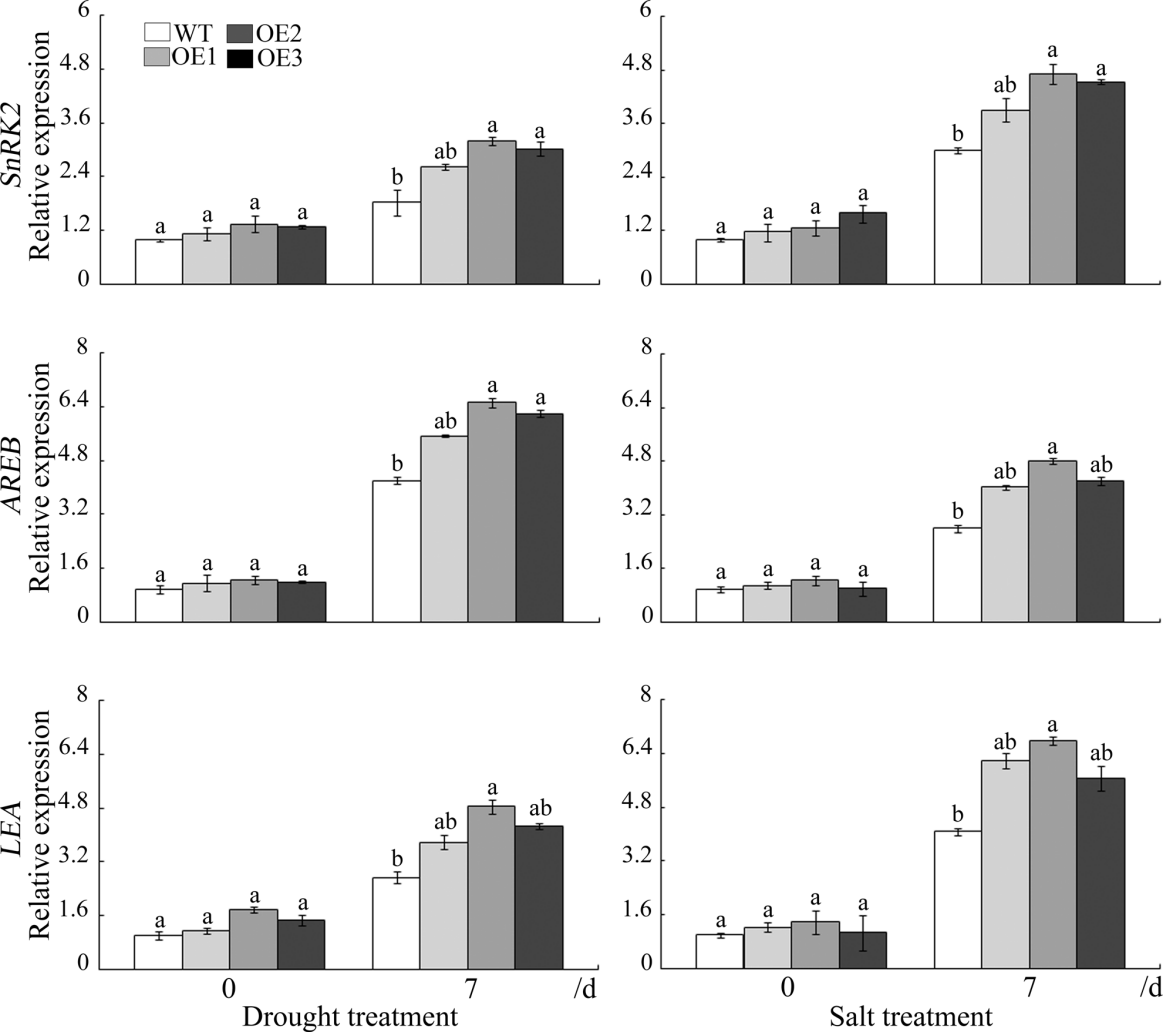
The expression of genes associated with ABA signaling in WT and OE plants following drought or salt stress.

It is widely known that ABA participates in the regulation of stomatal movement. To explore whether *GhWRKY41*-dependent stomatal closure is related to ABA, we analyzed stomatal movement with or without ABA treatment. We found that ABA treatment for 2.5 h reduced the stomatal apertures of the WT and OE lines (Fig 9A and 9B). This reduction was more severe in the OE lines. In addition, the expression of *rbohA* and *rbohB*, which are reported to be involved in ABA signaling, was upregulated in the OE lines under drought stress (Fig 9C). The expression of genes associated with ABA signaling, including *SnRK2* (*SNF1-related protein kinase 2*), *AREB* (*ABA-responsive element binding protein*) and *LEA* (*late embryogenesis abundant protein*), was also analyzed under drought and salt stress. As shown in Fig 10, the expression of these genes was significantly induced by drought or salt treatment. These results indicated that ABA participates in *GhWRKY41*-induced stomatal closure.

## Discussion

WRKY transcription factors constitute a large family in plants. Members of this family have been reported to be involved in various stress-related responses [28, 29]. However, the functions of WRKY proteins in non-model plants have remained unclear. In the present study, *GhWRKY41* was isolated from cotton, a crop with critical economic importance. One WRKY domain and a typical group III C_2_HC zinc finger domain [30] were present in the *GhWRKY41* sequence. Amino acid comparisons and phylogenetic analysis indicated that GhWRKY41 showed high similarity with PtWRKY12, BgWRKY, GmWRKY41 and AtWKRY41, all of which belong to the group III WRKY family. These results indicated that GhWRKY41 is a group III WRKY transcription factor.

The WRKY domain present in WRKY proteins can bind to W-box *cis*-acting elements that are mainly located in PR proteins. For this reason, WRKY proteins have been rigorously analyzed to determine their function in the plant defense response [31]. Moreover, Kalde *et al*. systematically analyzed the response of 13 *Arabidopsis* group III WRKY genes to SA and pathogens, and the results demonstrated that the majority of this subgroup could be upregulated by these treatments [32]. In our study, *GhWRKY41* exhibited significant changes in response to osmotic stresses. This observation may indicate that GhWRKY41 has a novel role as a member of the group III family of proteins.

In addition to their roles in biotic resistance, the roles of WRKY proteins in abiotic tolerance have also been studied [33, 34]. The expression of *GhWRKY41* was induced by osmotic stresses such as salt and drought. This induction is similar to that of *BcWRKY46* and *DgWRKY3* [35, 36]. Moreover, the *in vivo* role of *GhWRKY41* in plant abiotic tolerance was clearly demonstrated in transgenic tobacco lines overexpression *GhWRKY41*. Under drought and salt stress conditions, WT plants were smaller, more withered and more chlorotic, while GhWRKY41-expressing tobacco plants were better able to adapt to these stresses. Similarly, constitutive expression of different WRKY genes, including *VpWRKY2* and *GsWRKY20*, also resulted in greater tolerance to abiotic stresses in transgenic lines [18, 37]. Therefore, we conclude that GhWRKY41 is a functional WRKY transcription factor involved in the response to salt and drought stress.

ROS such as superoxide, hydrogen peroxide and hydroxyl radicals play a dual role in plants: they act as necessary signaling molecules, but they can cause damage to plant cells when overproduced under stress conditions [38]. Plants must maintain a ROS balance to minimize cellular damage caused by stress. Among the variety of ROS compounds, the present study investigated the effects of H_2_O_2_ in abiotic stress-resistant plants. Our study found that *GhWRKY41*-overexpressing plants exhibited lower H_2_O_2_ accumulation than WT plants under drought and salt stress conditions. MDA is widely recognized as a parameter that reflects lipid peroxidation [39]. *GhWRKY41* might protect plants by promoting the degradation of MDA. Our results showed that overexpression of *GhWRKY41* reduced the accumulation of H_2_O_2_ in transgenic seedlings. Moreover, the expression of *GhWRKY41* was induced by treatment with H_2_O_2._ Consequently, we infer that overexpression of *GhWRKY41* in tobacco confers drought and salt stress tolerance by promoting ROS elimination.

When plants exposed to stress overproduce ROS, they must develop tactics to scavenge ROS and protect cells. Enzymes such as SOD, CAT and POD act as ROS scavengers. An analysis of these enzymes showed that overexpression of *GhWRKY41* markedly strengthened their activity under drought and salt stresses. At the molecular level, the expression of genes encoding these enzymes was also upregulated under stress. Thus, overexpression of *GhWRKY41* may regulate these genes and induce the activity of a more efficient antioxidant system to counteract the oxidative stress evoked by salt and drought stress.

ABA acts as an important regulator and plays a complex role in abiotic stress signaling [40]. Various environmental stresses result in the rapid accumulation of ABA, leading to stomatal closure and the inhibition of stomatal opening, which reduces water loss by transpiration [41]. It has been reported that reduction of the water loss rate is a major factor contributing to drought tolerance, and transpirational water loss through the stomata is a key determinant of drought tolerance [19]. Li *et al*. reported that *AtWRKY54* and *AtWRKY70* enhanced tolerance to osmotic stress by regulating stomatal aperture. Salt stress is often accompanied by drought stress, and both stresses cause water deprivation through ABA-dependent and ABA-independent pathways [42]. A recent study showed that transient alkalinization, a remote effect of chloride stress, modulates the compartmental distribution of ABA between the leaf apoplast and the guard cells and is instrumental in inducing stomata closure during the initial stages of salt stress [43]. In our study, compared to the WT line, the transgenic lines showed enhanced stomatal closure under drought stress, salt stress and ABA treatment. ABA controls stomatal movement via a dual mechanism. Regulation can occur via the biochemical effects of ABA on guard cells, or it can occur via a decrease in water content in leaf vascular tissues [44]. Respiratory burst oxidase homolog proteins (rbohs) are NAD(P)H oxidase homologues that have a direct regulatory effect on Ca^2+^ and on the activity of the NADPH oxidase in plants [45]. Many rbohs can promote ABA-induced stomatal closing, ROS production, ABA-induced cytosolic Ca^2+^ increases and ABA activation of plasma membrane Ca^2+^-permeable channels in guard cells in *Arabidopsis* [46]. The GUS staining assay indicated that salt and drought stresses enhanced *GhWRKY41* expression in guard cells. The upregulation of *rbohA* and *rbohB* expression in the OE lines under drought stress indicated that the overexpression of *WRKY41* could enhance rbohA and rbohB activities to upregulate ROS production in the OE lines, thereby enhancing stomatal closure. However, darker DAB staining was observed in the WT lines. This result may be caused by antioxidant enzymes. These data suggest that *GhWRKY41* acts as a positive regulator of stomatal closure in an ABA-dependent manner.

In summary, this study identified *GhWRKY41* as a positive regulator of salt and drought stress responses. Overexpression of *GhWRKY41* in tobacco improved plant tolerance to salt and drought stresses. This enhanced drought/salt tolerance in OE transgenic plants was associated with enhanced expression of several known stress-responsive genes and enhanced stomata closure, suggesting that overexpression of *GhWRKY41* may lead to better osmotic adjustment, reduced membrane damage, and minimized oxidative stress. It should be noted that the function of *GhWRKY41* in drought and salt tolerance must be directly addressed in cotton plants in the future. Additional work is also necessary to understand the molecular mechanisms of *GhWRKY41* in salt and drought stress responses.

## Supporting Information

S1 Table Oligonucleotide primers used in cloning and vector construction. (DOC)

S2 Table Oligonucleotide primers used in qPCR. (DOC)

S3 Table Putative *cis*-acting elements in the *GhWRKY41* upstream fragment. (DOC)

S1 Fig Characterization and sequence analysis of GhWRKY41. (A) Alignment of the deduced GhWRKY41 protein sequence with other known WRKY homologs proteins. Identical amino acids are highlighted in blue. The WRKYGQK amino acids are boxed. The C and H residues in the zinc-finger motif are marked by a *triangle*. The nuclear localization signal (NLS), KKRK, is marked by an asterisk. (B) Phylogenetic analysis of the GhWRKY41 protein.
(TIF)

S2 Fig RT-PCR of transgenic plants. (A) Evaluation of *GhWRKY41* expression in the T_1_ progeny of transgenic plants. (B) Evaluation of *GhWRKY41* expression in the T_3_ progeny of three independent transgenic lines.
(TIF)

S3 Fig Relative water loss rates from detached leaves of WT and transgenic plants were measured each hour. (TIF)
