# Correction: The Cotton WRKY Gene *GhWRKY41* Positively Regulates Salt and Drought Stress Tolerance in Transgenic *Nicotiana benthamiana*

**DOI:** 10.1371/journal.pone.0157026

**Published:** 2016-06-02

**Authors:** Xiaoqian Chu, Chen Wang, Xiaobo Chen, Wenjing Lu, Han Li, Xiuling Wang, Lili Hao, Xingqi Guo

The authors would like to correct Figs 6 and 8, as errors were introduced in the preparation of these figures for publication. In Figs 4A and 6A, the same image was used to represent the MS control in both figures. The authors have provided a corrected Fig 6 here with a new MS image from another biological replicate. In Fig 8A, the same image was inadvertently used to represent both the WT Control and OE2 Control conditions. The authors have provided a corrected Fig 8 here with the correct OE2 Control image. The authors confirm that these changes do not alter their findings and have provided the underlying images for Fig 8 as Supporting Information.

**Fig 6:**
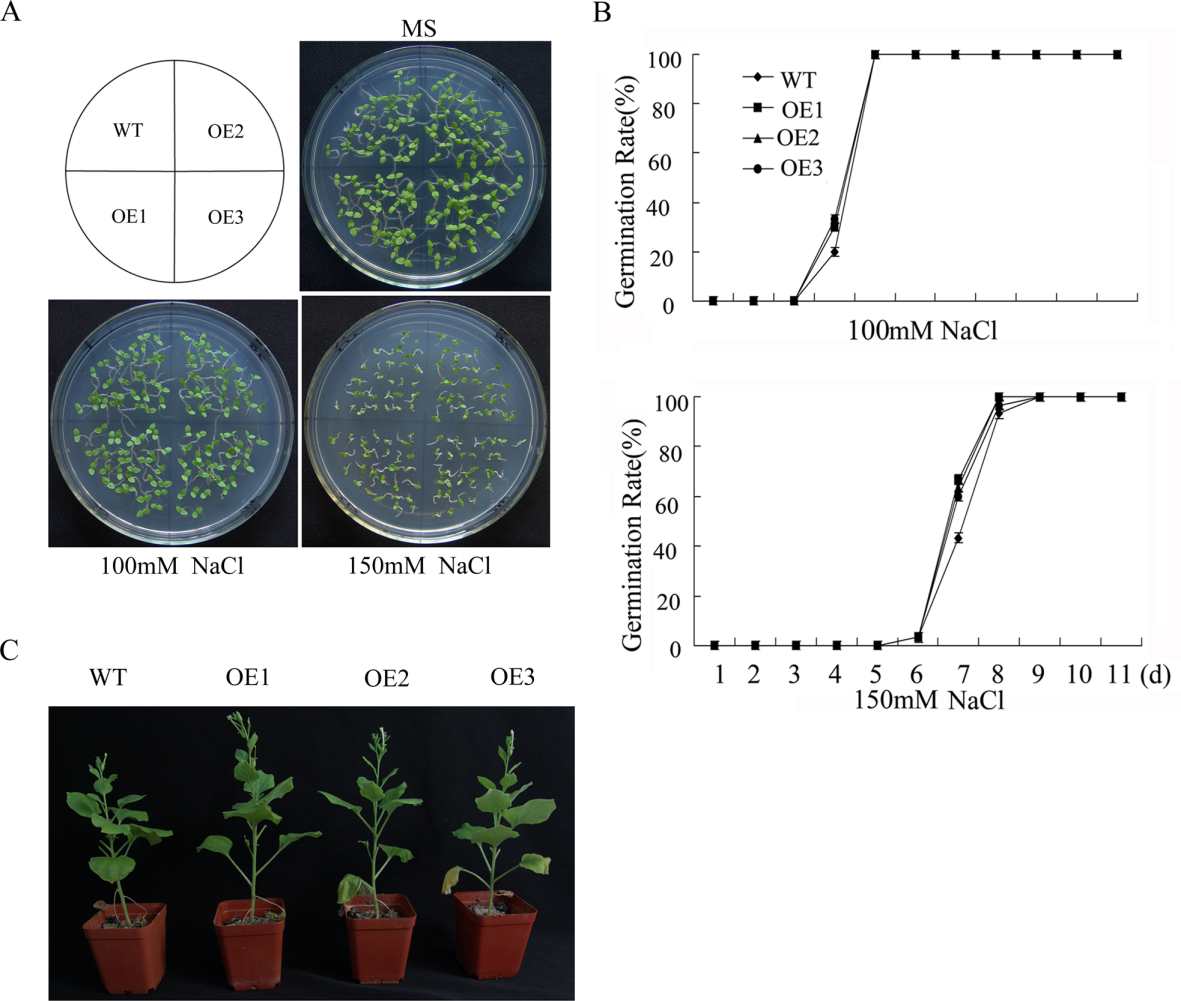
Salt tolerance of WT and *GhWRKY41*-overexpressing *N*. *benthamiana* plants. (A, B) Seed germination assay. (C) Photograph of representative 8-week-old WT and OE plants watered with 200 mM NaCl for 1 month.

**Fig 8:**
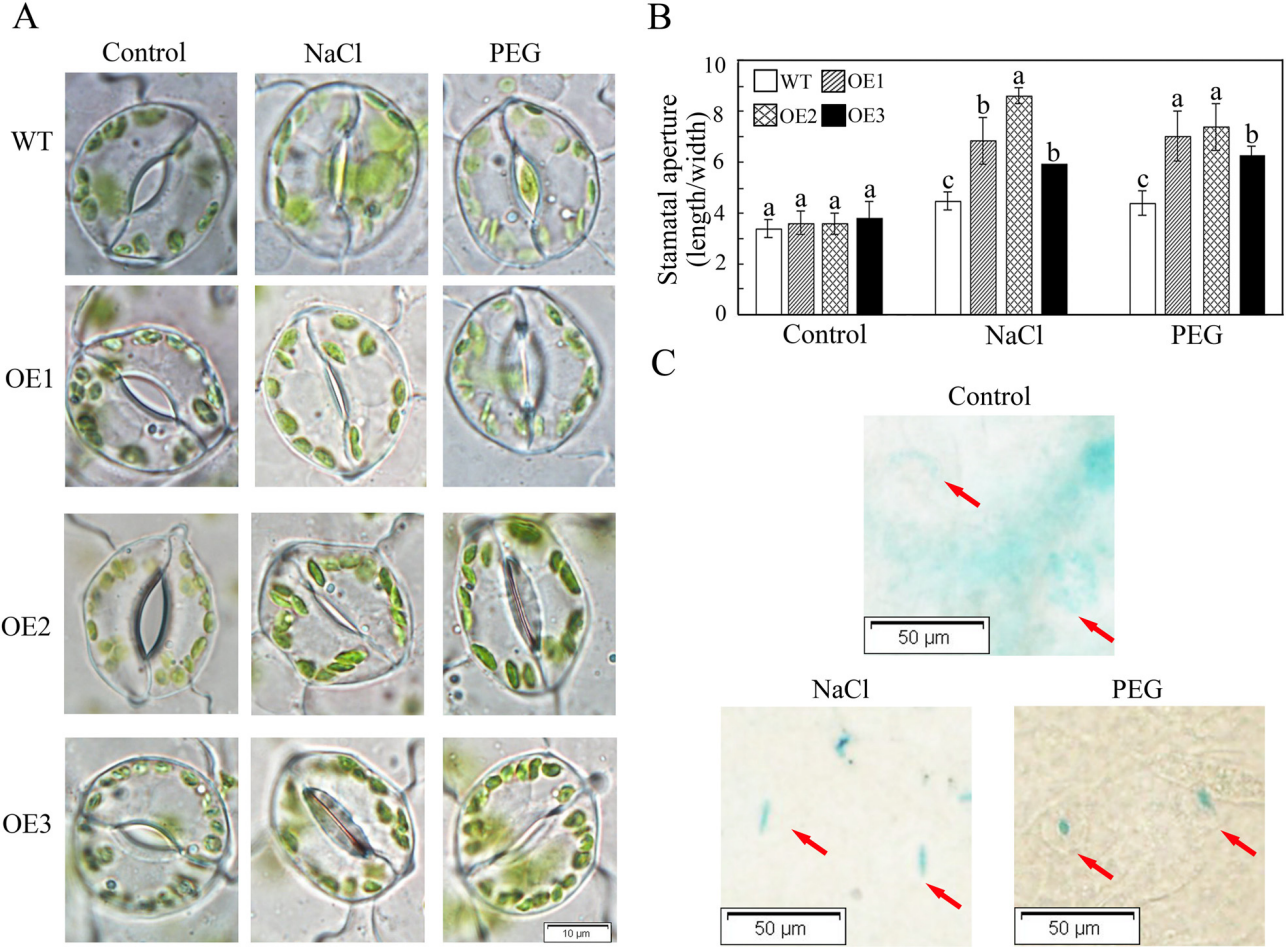
*GhWRKY41* regulates stomatal movement. (A) Comparison of stomatal aperture in response to salt and drought. (B) Stomatal aperture data were calculated from 50 stomata from the leaves of three different plants. Values are the mean ± SD. (C) GUS staining of leaves from transgenic *Arabidopsis* exposed to salt and drought treatments.

## Supporting Information

S1 File Raw images used to create Fig 8. (ZIP)
