# Supplementary material for: The Cotton WRKY Gene GhWRKY41 Positively Regulates Salt and Drought Stress Tolerance in Transgenic Nicotiana benthamiana
Source: PLoS One. 2015 Nov 12;10(11):e0143022. doi: 10.1371/journal.pone.0143022 (PMC4643055; doi:10.1371/journal.pone.0143022)
Supplement: S1 Table — (DOC) [file pone.0143022.s001.doc]

**Table S1.** The primers used in this study

| Primer | Primer sequence (5′-3′) |
| --- | --- |
| Internal degenerate primers (D=A,G or T; N=A, C, G or T; R=A or G; V=A,C, or G; Y=C or T) | |
| M1 | CTCAAAGAARAGAAAGAC |
| M2 | ADGAGAAAAATCCWGGKGT |
| 5' RACE primers | |
| 5P1 | TTGGGTGTTTCGATAGGTG |
| 5P2 | TAGCTTCTAGGATATTTGGCTC |
| 5P3 | TCTAAATCATCACTCCTGGGGC |
| 5P4 | GGCACACAAGTTATTCCTCCTACAT |
| AAP | GGCCACGCGTCGACTAGTAC(G)16 |
| AUAP | GGCCACGCGTCGACTAGTAC |
| 3' RACE primers | |
| 3P1 | TGTCTCCCGATACACCAG |
| 3P2 | GTTTCAGCCAACACTTCGG |
| B26 | GACTCTAGACGACATCGA(T)18 |
| B25 | GACTCTAGACGACATCGA |
| The full-length cDNA primers | |
| Q1 | CTTACAGTGGAAGGAAAGAAGA |
| Q2 | TGAAATGAAAGGGAGATGTATTGT |
| Primers used in expression vector | |
| Z1 | TCTAGAATGGAGAACATGTGGAAGTGGGAGC |
| Z2 | GTCGACCGCAGAACACACGGCTCACC |
| N1 | TCTAGAATGGAGAACATGTGGAAGTGGGAGC |
| N2 | CTCGAGGGAGAAAAATCCCGGGGTGTC |
| Y1 | CCATGGAGATGGAGAACATGTGGAAGTGGG |
| Y2 | GGATCCGGAGAAAAATCCCGGGGTGTC |
| 35SF | TCAGAAAGAATGCTAACCCACAG |
| 35SR | GAAGGGTCTTGCGAAGGATAG |
| Primers used in promoter isolation | |
| Tail1 | CCTTTTCTAAATCATCACTCCTGGGGCTG |
| Tail2 | CGTAGGCCTGCTCAGTTTCAAAATCAG |
| Tail3 | CATTCCTTGAATCAGCTCGCTCACC |
| Tail4 | CCGAGTTTTTCAGCATAGACTAGTC |
| Tail5 | GTGGACTGAAGAGTGAGGTAAGGTC |
| Tail6 | CACAAATGCTTATAAACCGAGATGAG |
| LAD1 | ACGATGGACTCCAGAGVNVNNNGGAA |
| LAD2 | ACGATGGACTCCAGAGBNBNNNGGTT |
| LAD3 | ACGATGGACTCCAGAGVVNVNNNCCAA |
| LAD4 | ACGATGGACTCCAGAGBDNBNNNCGGT |
| AC1 | ACGATGGACTCCAGAG |
| P1 | GGTACCAGTATAACCAACAGTCCAATGAG |
| P2 | GGATCCTTTTCTTTTCCTTTTCTTCTTTCC |
