# Supplementary material for: The Cotton WRKY Gene GhWRKY41 Positively Regulates Salt and Drought Stress Tolerance in Transgenic Nicotiana benthamiana
Source: PLoS One. 2015 Nov 12;10(11):e0143022. doi: 10.1371/journal.pone.0143022 (PMC4643055; doi:10.1371/journal.pone.0143022)
Supplement: S2 Table — (DOC) [file pone.0143022.s002.doc]

| Primer | Primer sequence (5´-3´) |
| --- | --- |
| *GhSSU* | AACTTAAAGGAATTGACGGAAG, forward |
|  | GCATCACAGACCTGTTATTGCC, reverse |
| *Gh18S rRNA* | AACTTAAAGGAATTGACGGAAG, forward |
|  | GCATCACAGACCTGTTATTGCC, reverse |
| *Nbβ-actin* | TTGTGTTGGACTCGGGTGATG, forward |
|  | GCTCATTCTATCGGCAATACCTG, reverse |
| *NtGST* | AGCACCCTTACCTTTCCCTC, forward |
|  | GCAGCTTCTCCAATCCCTTAAC, reverse |
| *NtCAT* | cacagccacgctactcaagac, forward |
|  | CCACCCACCGACGAATAAAG, reverse |
| *NbAPX* | GGAGTGGTTGCTGTTGAAGTC, forward |
|  | GGAGAGCCTTGTCTGATGG, reverse |
| *NtSOD* | caactccacggcttccagac, forward |
|  | TGGGTCCTGATTAGCAGTGGT, reverse |
| *NbrbohA* | ACACACGCCATCAGAACTCCA, forward |
|  | CCCACCCAACCAAAATACGC, reverse |
| *NbrbohB* | GTTTGCCAGCCACCACCTAAT, forward |
| *NbAREB*  *NbSnRK2.3*  *NbLEA* | AAGAGCAGAACGAGCATCACC, reverse  TTGCTGGTGGAAATGTAAGTGC, forward  GGAATGTAACATCCTTTGAGTATCG, reverse  GGCTAGGCTAGTTAAGGACAAGA, forward  GTTCTCCACCTGCTGCATACTCC, reverse  GGATCTAATTGACAAGGCGAAG, forward  CTCGCCGCTATAAGAGAGAG, reverse |

**Table S2. Oligonucleotide primers used in qPCR**
