# Supplementary material for: The Cotton WRKY Gene GhWRKY41 Positively Regulates Salt and Drought Stress Tolerance in Transgenic Nicotiana benthamiana
Source: PLoS One. 2015 Nov 12;10(11):e0143022. doi: 10.1371/journal.pone.0143022 (PMC4643055; doi:10.1371/journal.pone.0143022)
Supplement: S3 Table — (DOC) [file pone.0143022.s003.doc]

| *cis*-element | Position | Sequence (5'-3') |
| --- | --- | --- |
| **Abiotic stress response elements** | | |
| MYBCORE | -93(+), -25(-) | CNGTTR |
| DRE1COREZMRAB17 | -593(-) | ACCGAGA |
| MYB1AT | -21(+), -1092(+) | WAACCA |
| MBS | -1029(-) | TAACTG |
| MYCCONSENSUSAT | -87(+), -359(+), -529(+), -607(+), -625(+), -689(+) | CANNTG |
| WBOXNTERF3 | -701(+), -748(+), -664(-), -683(-), -789(-), -1118(-) | TGACY |
| **Pathogen/elicitor response elements** | | |
| WBOXATNPR1 | -700(+), -55(-), -790(-)764(+) | TTGAC |
| GT1GMSCAM4 | -770(+), -141(-), -823(-), -1079(-) | GAAAAA |
| WRKY71OS | -701(+),- 748(+), -55(-), -665(-), -684(-), -790(-), -1119(-) | TGAC |
| **Tissue-specific and development-related elements** | | |
| DPBFCOREDCDC3 | -624(+) | ACACNNG |
| GATABOX | -331(+),-401(+),-653(+),-857(-),-1009(-),  -617(-),-486(-),-263(-),-155(-),-116(-) | GATA |
| OSE2ROOTNODULE | CTCTT -424(+), -714(+), -915(+), -1016(+) | CTCTT |
| TAAAG motif | -370(+), -853(-) | TAAAG |
| POLLEN1LELAT52 | -668(+), -1188(+), -1199(+), -781(-), -804(-), -825(-), -876(-), -1081(-), -1166(-) | AGAAA |
| **Light regulation elements** | | |
| -10PEHVPSBD | -733(+) | TATTCT |
| GATA box | -102(+), -374(+), -632(+), -118(-), -449(-), -526(-), -743(-) | GATA |
| IBOXCORE | -117(-), -448(-), -742(-) | GATAA |
| INRNTPSADB | -710(+), -144(+), -717(+), -871(+), -899(+), -1036(+), -647(-) | YTCNATYY |
| **Auxin response elements** | | |
| ARFAT | -42(+) | TGTCTC |
