# Supplementary figures and images for: The Cotton WRKY Gene GhWRKY41 Positively Regulates Salt and Drought Stress Tolerance in Transgenic Nicotiana benthamiana
Source: PLoS One. 2015 Nov 12;10(11):e0143022. doi: 10.1371/journal.pone.0143022 (PMC4643055; doi:10.1371/journal.pone.0143022)

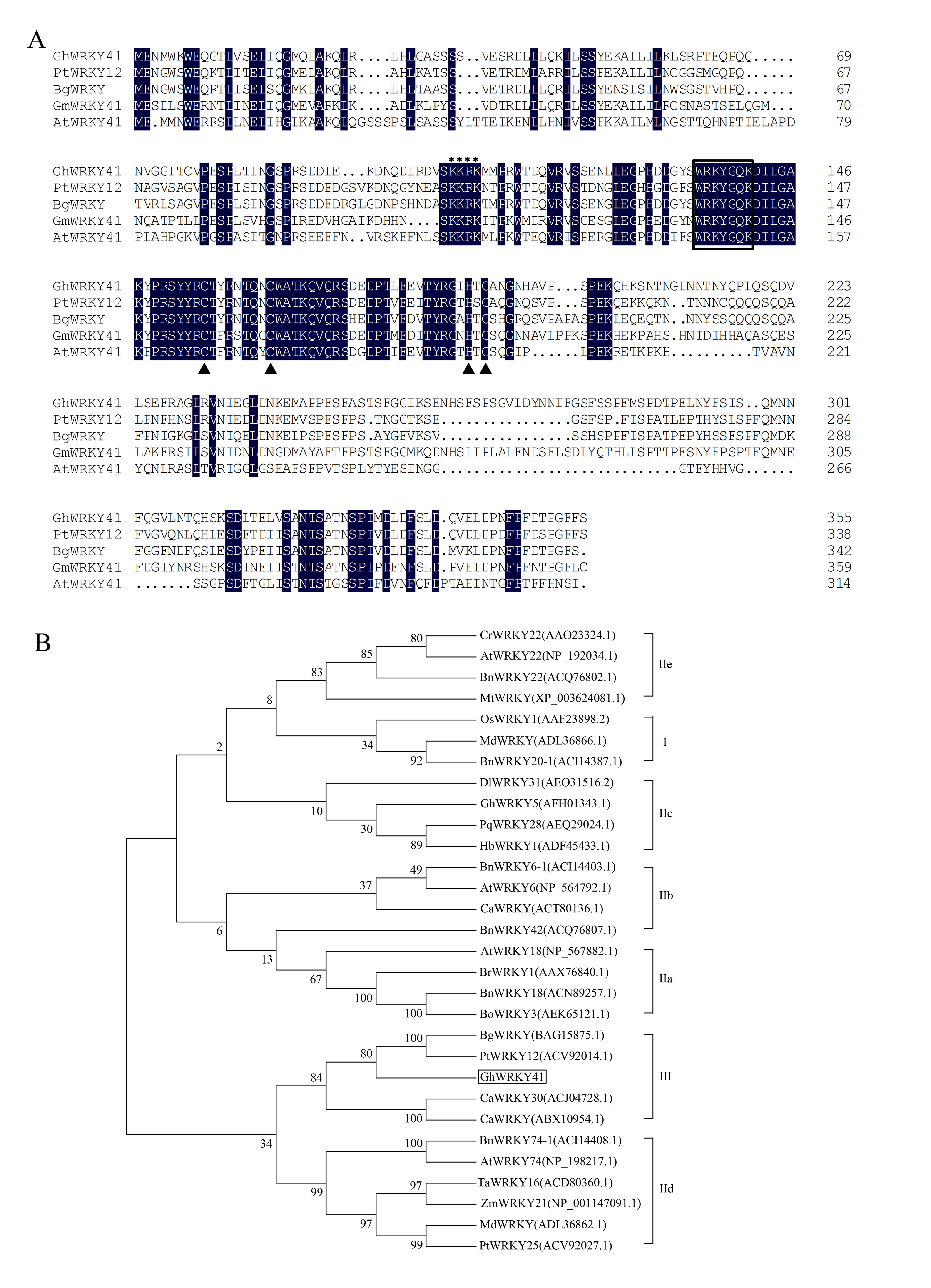

Supplement: S1 Fig — (A) Alignment of the deduced GhWRKY41 protein sequence with other known WRKY homologs proteins. Identical amino acids are highlighted in blue. The WRKYGQK amino acids are boxed. The C and H residues in the zinc-finger motif are marked by a triangle. The nuclear localization signal (NLS), KKRK, is marked by an asterisk. (B) Phylogenetic analysis of the GhWRKY41 protein. (TIF) [file pone.0143022.s004.tif]

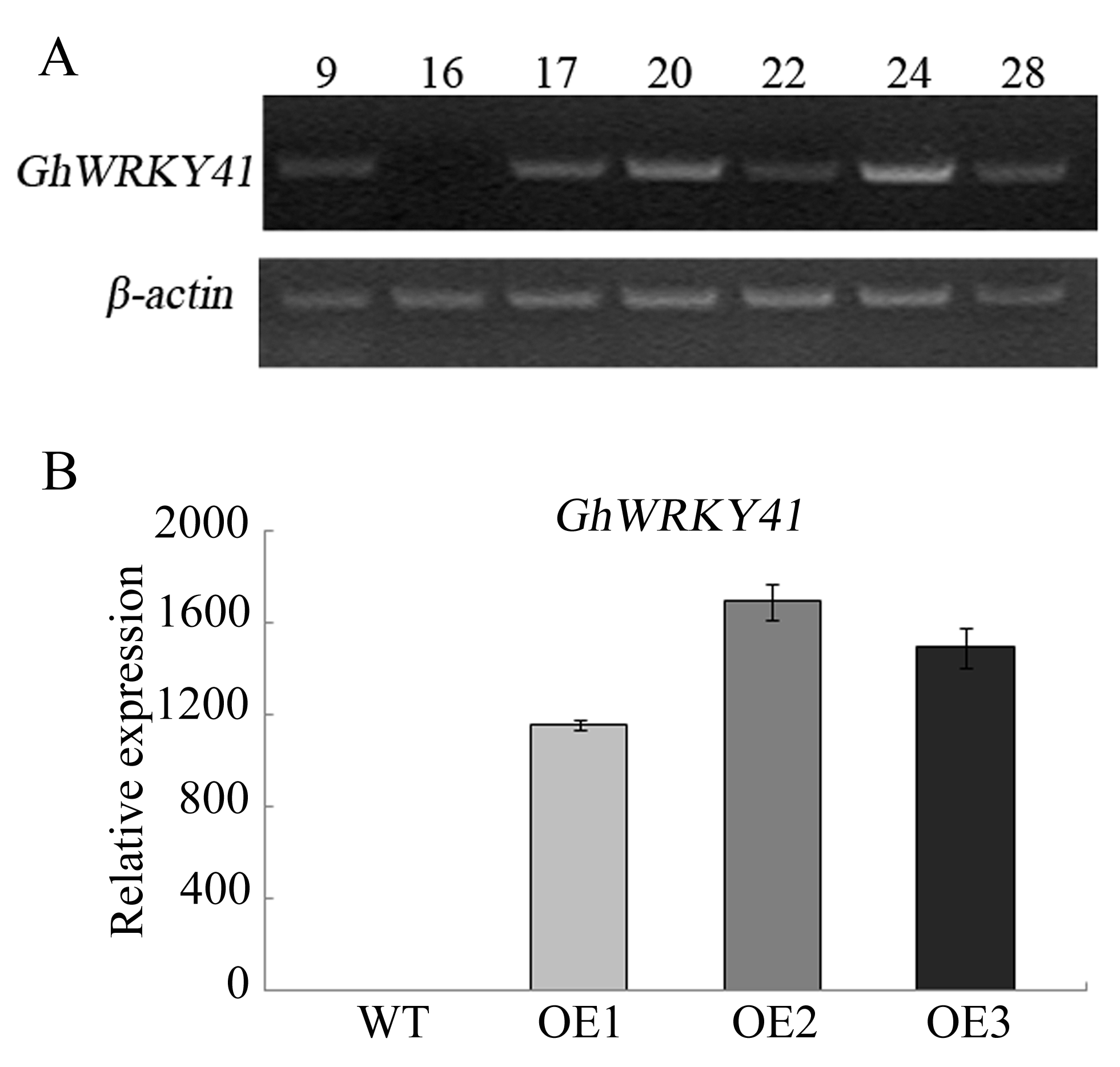

Supplement: S2 Fig — (A) Evaluation of GhWRKY41 expression in the T1 progeny of transgenic plants. (B) Evaluation of GhWRKY41 expression in the T3 progeny of three independent transgenic lines. (TIF) [file pone.0143022.s005.tif]

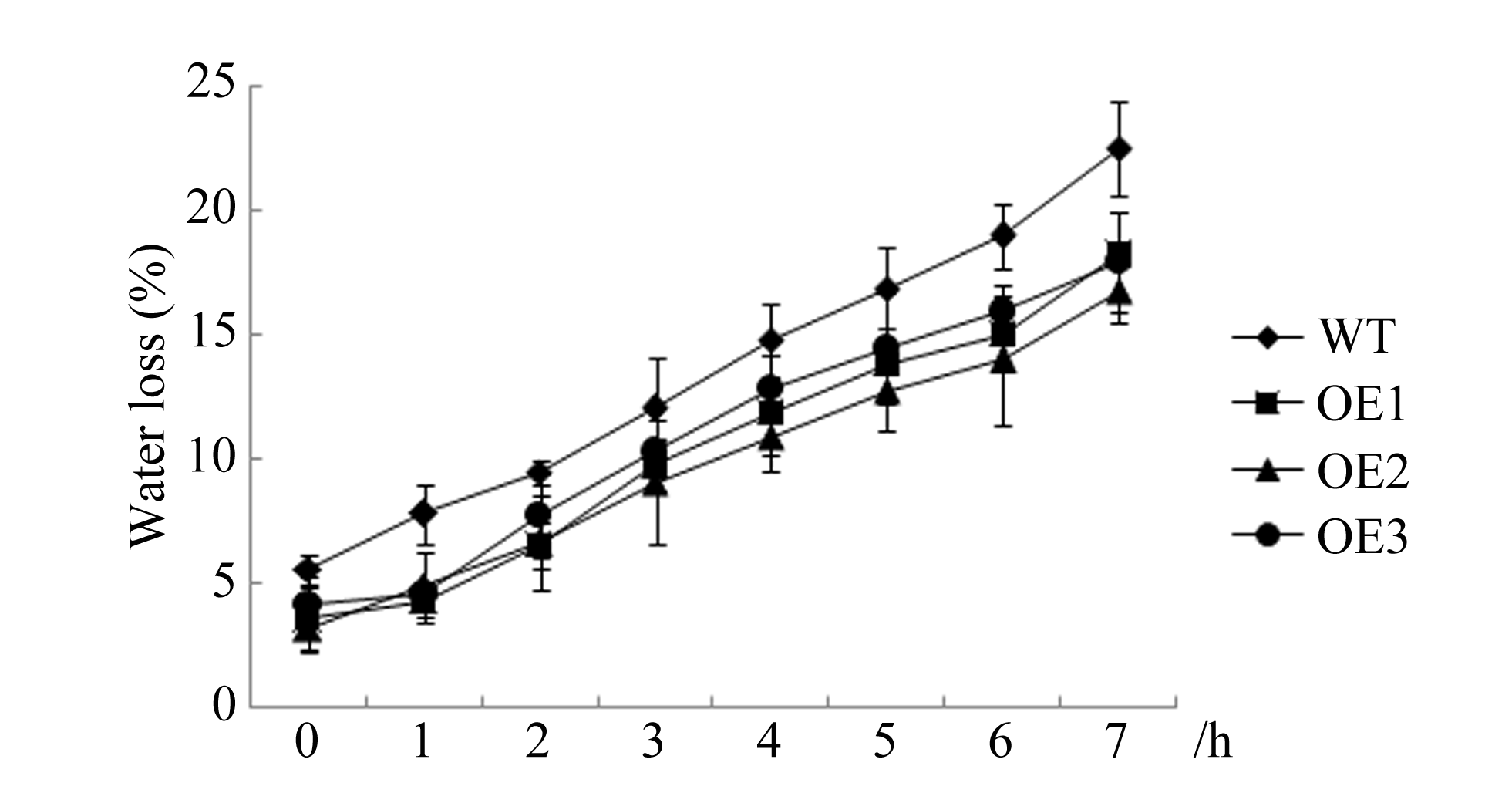

Supplement: S3 Fig — (TIF) [file pone.0143022.s006.tif]
