# Supplementary figures and images for: Correction: The Cotton WRKY Gene GhWRKY41 Positively Regulates Salt and Drought Stress Tolerance in Transgenic Nicotiana benthamiana
Source: PLoS One. 2016 Jun 2;11(6):e0157026. doi: 10.1371/journal.pone.0157026 (PMC4890847; doi:10.1371/journal.pone.0157026)

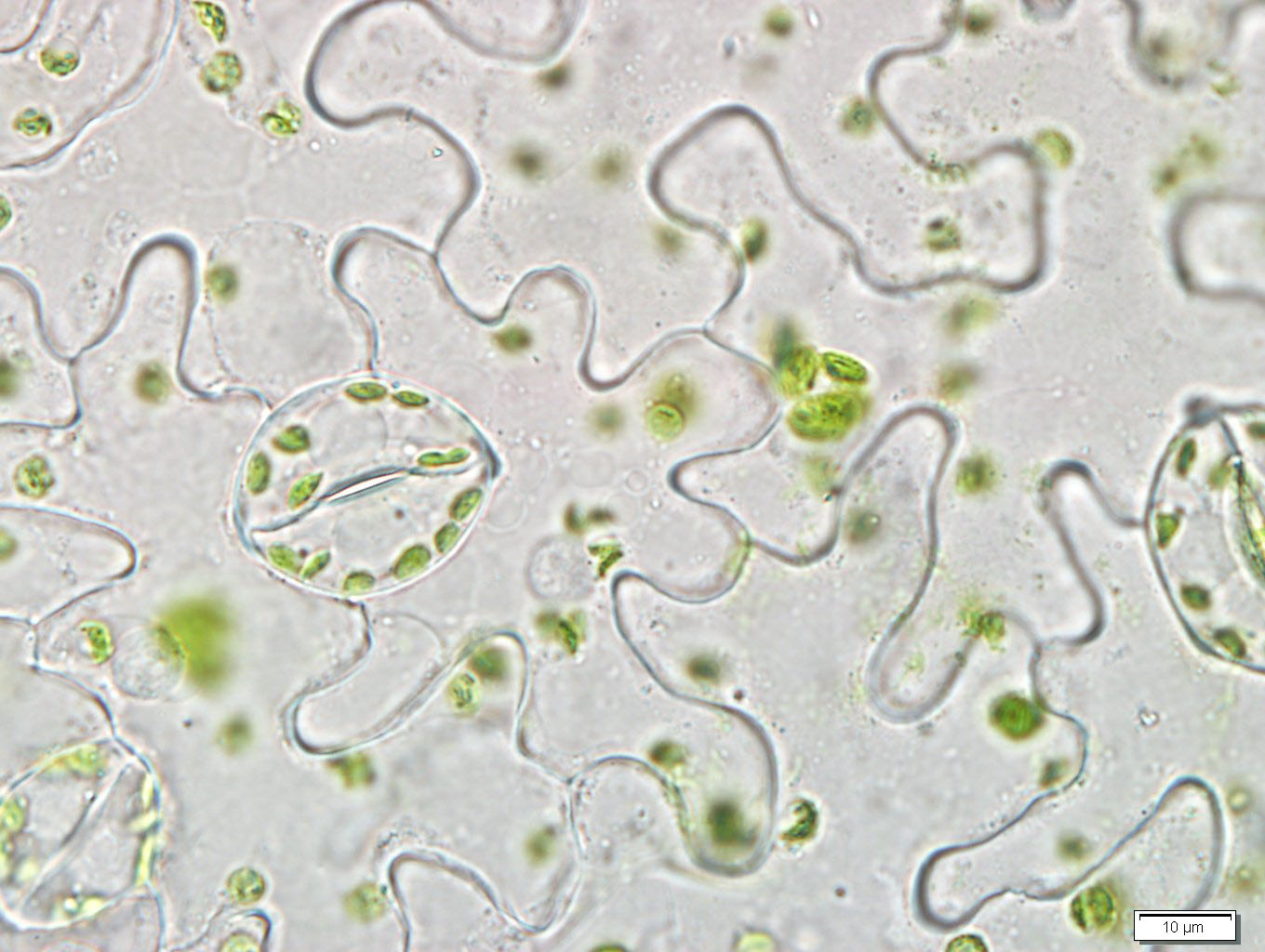

Supplement: S1 File — (ZIP) [file pone.0157026.s001.zip › FIG8/FIG8 OE1 NaCl.jpg]

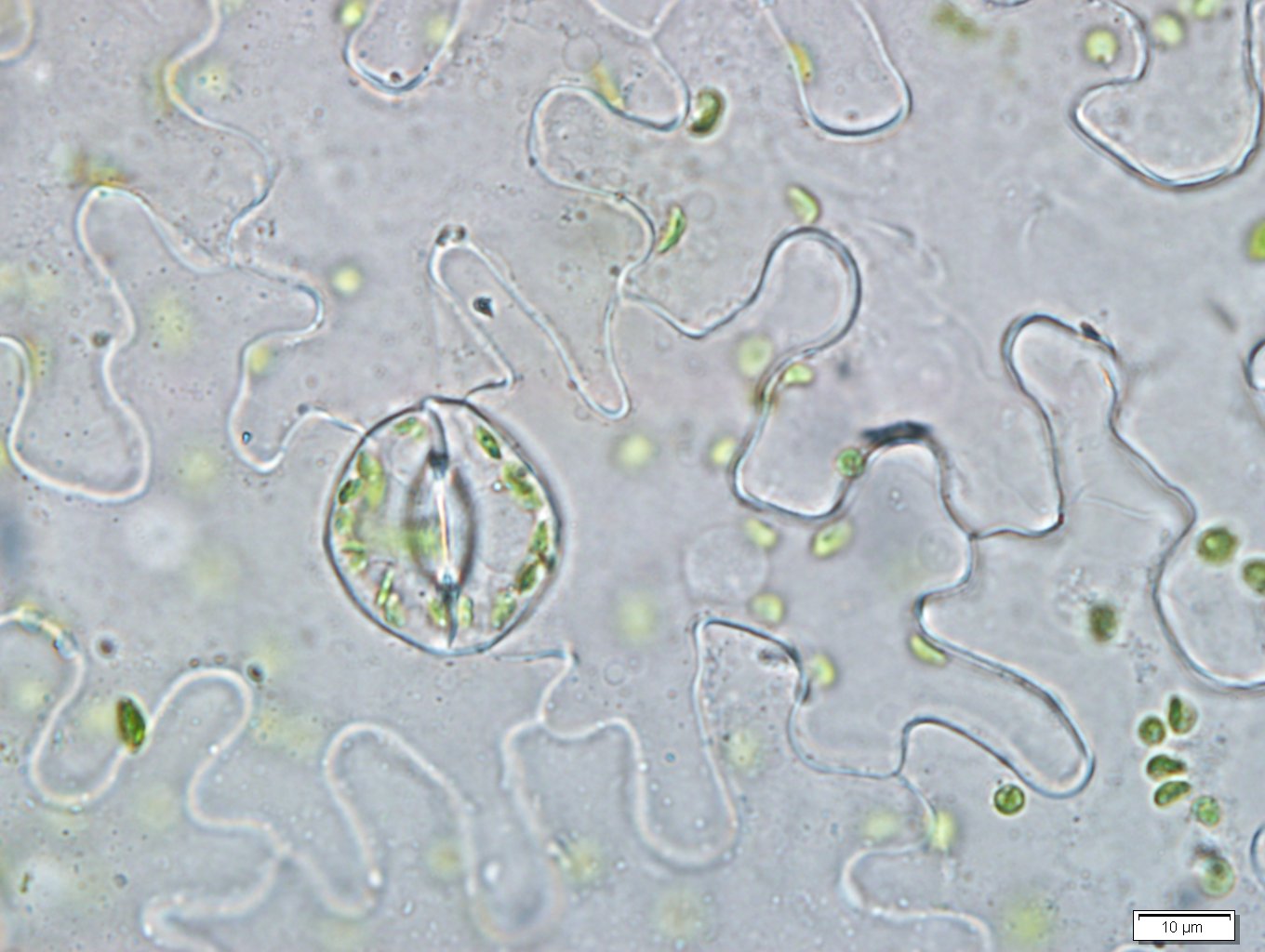

Supplement: S1 File — (ZIP) [file pone.0157026.s001.zip › FIG8/FIG8 OE1 PEG.jpg]

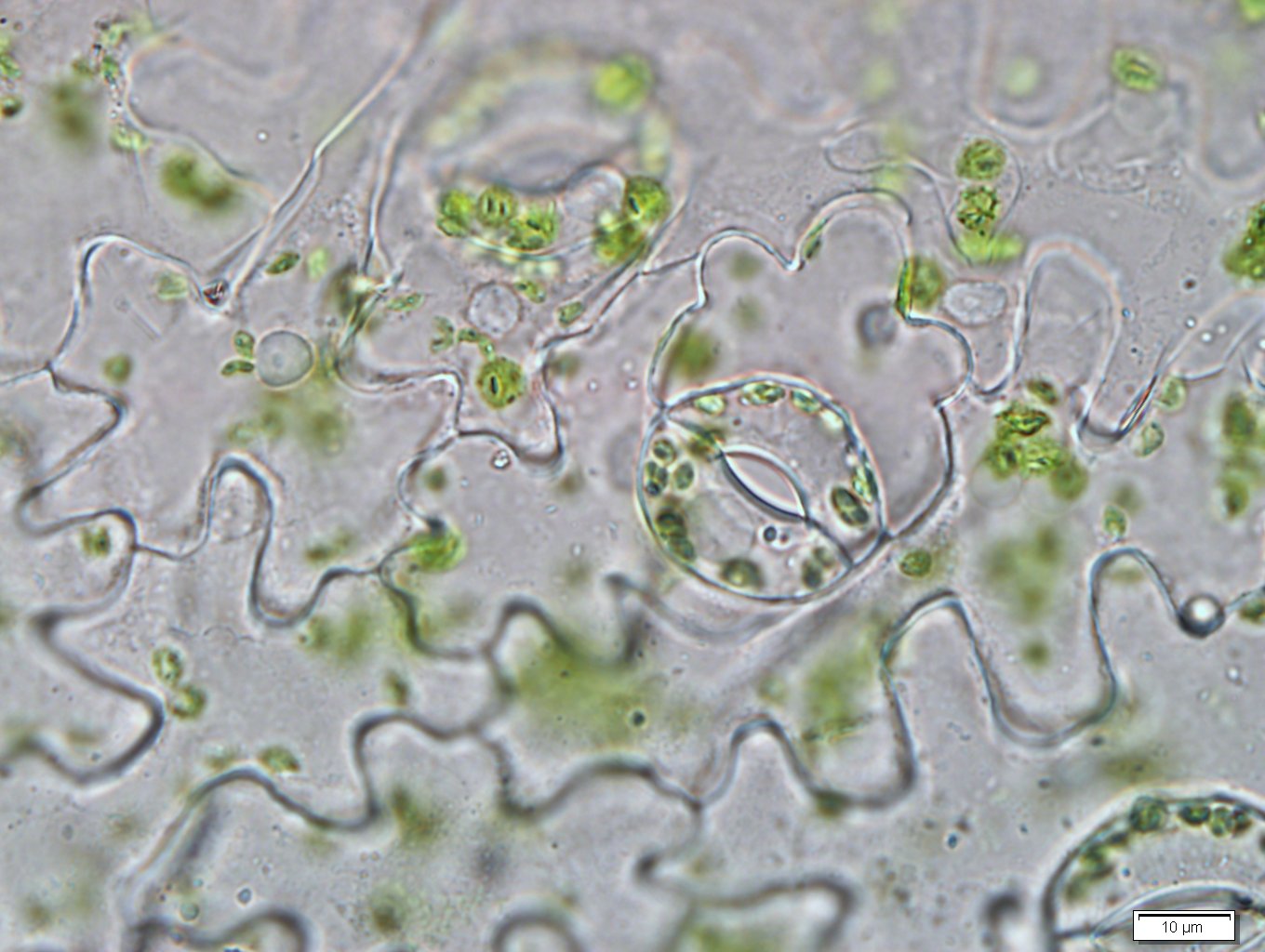

Supplement: S1 File — (ZIP) [file pone.0157026.s001.zip › FIG8/FIG8 OE1 control.jpg]

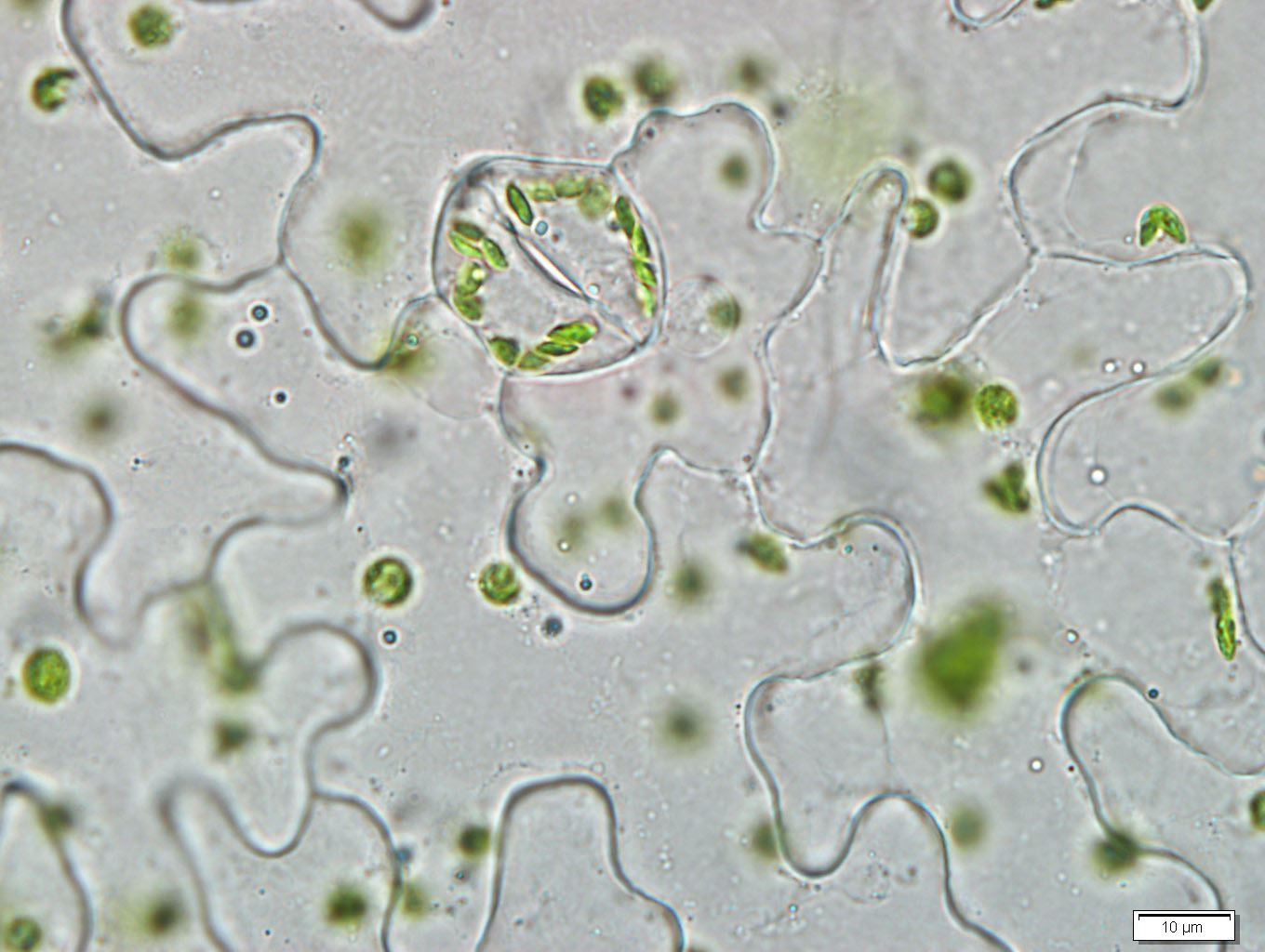

Supplement: S1 File — (ZIP) [file pone.0157026.s001.zip › FIG8/FIG8 OE2 NaCl.jpg]

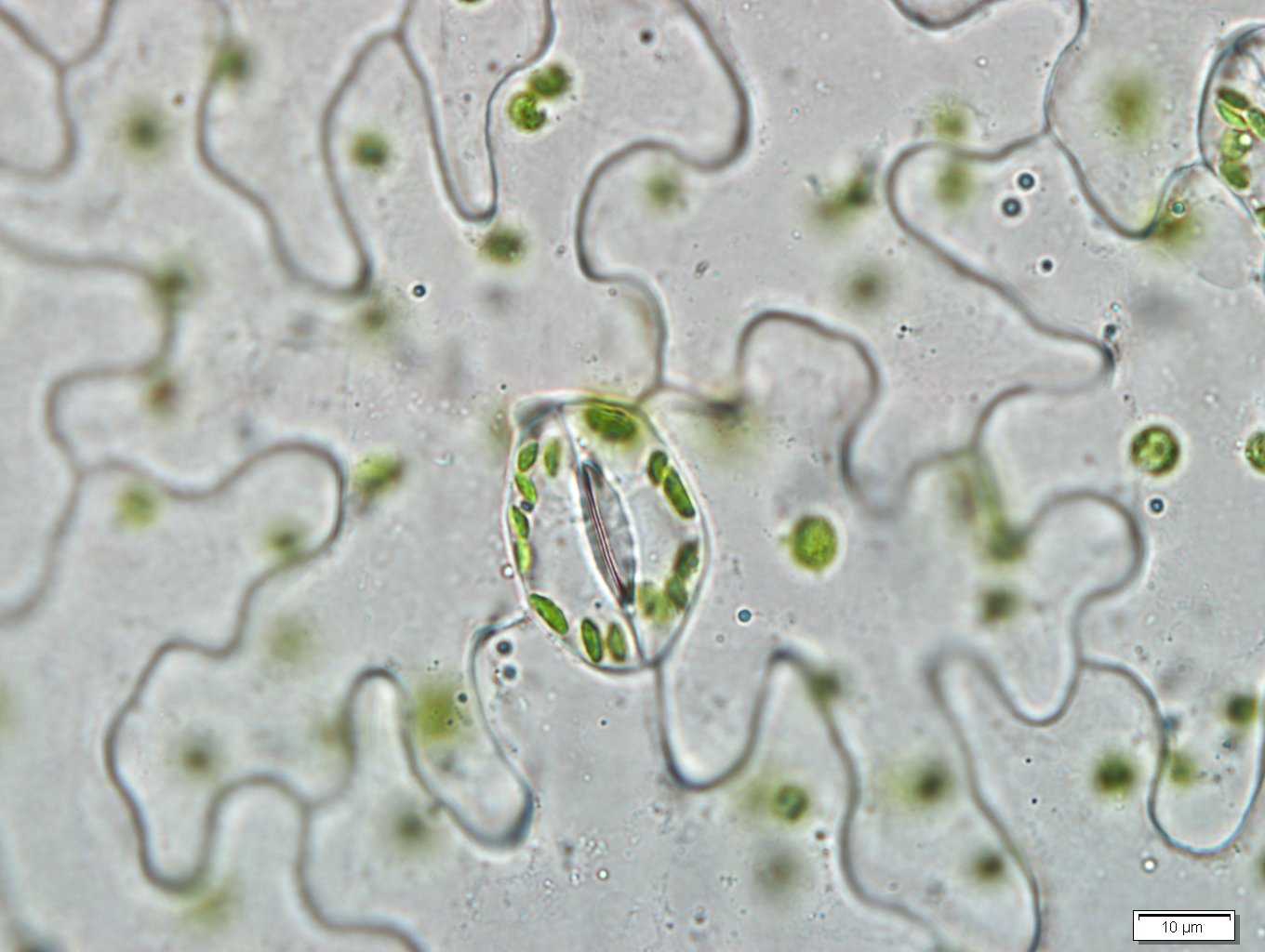

Supplement: S1 File — (ZIP) [file pone.0157026.s001.zip › FIG8/FIG8 OE2 PEG.jpg]

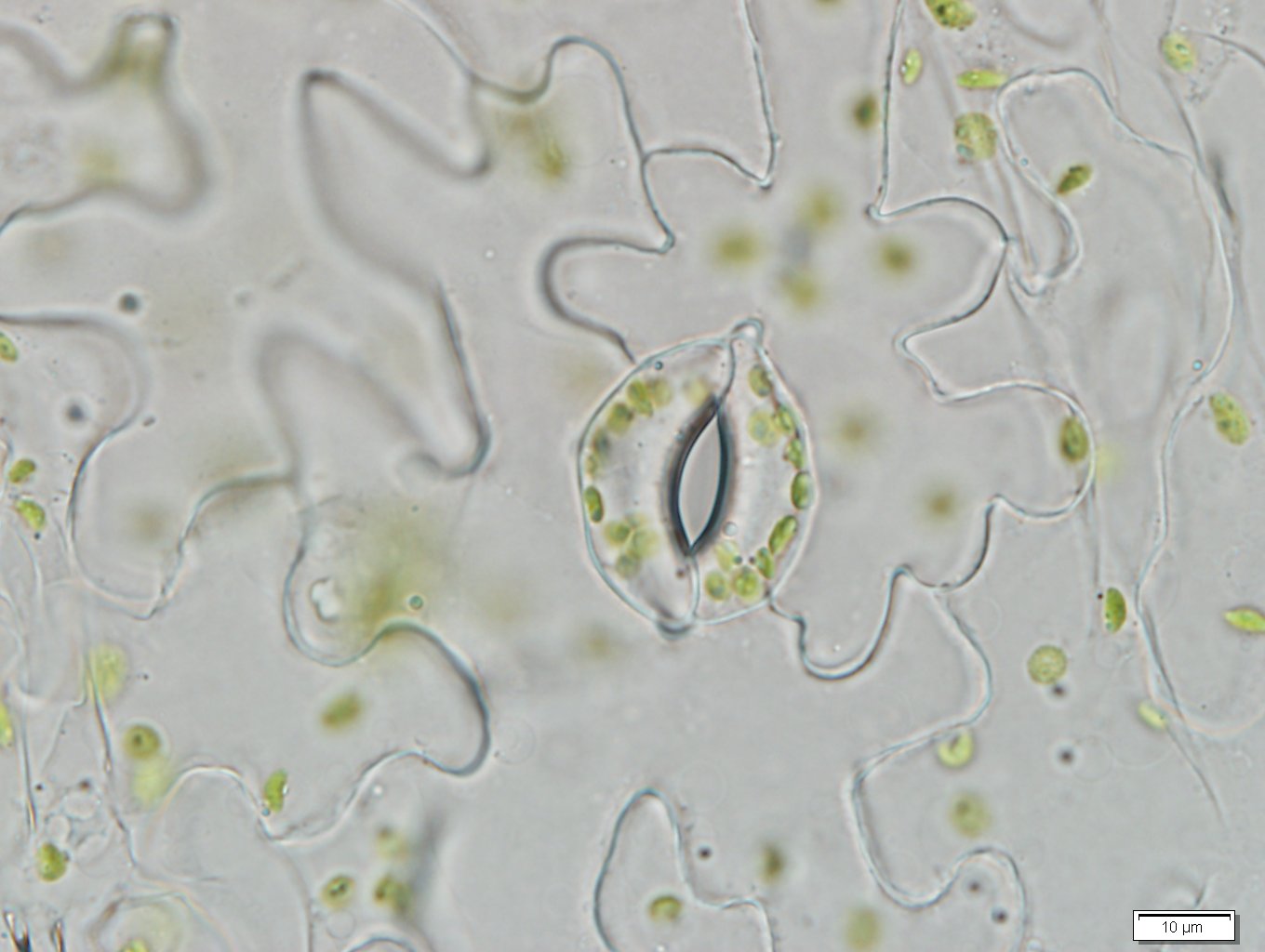

Supplement: S1 File — (ZIP) [file pone.0157026.s001.zip › FIG8/FIG8 OE2 control.jpg]

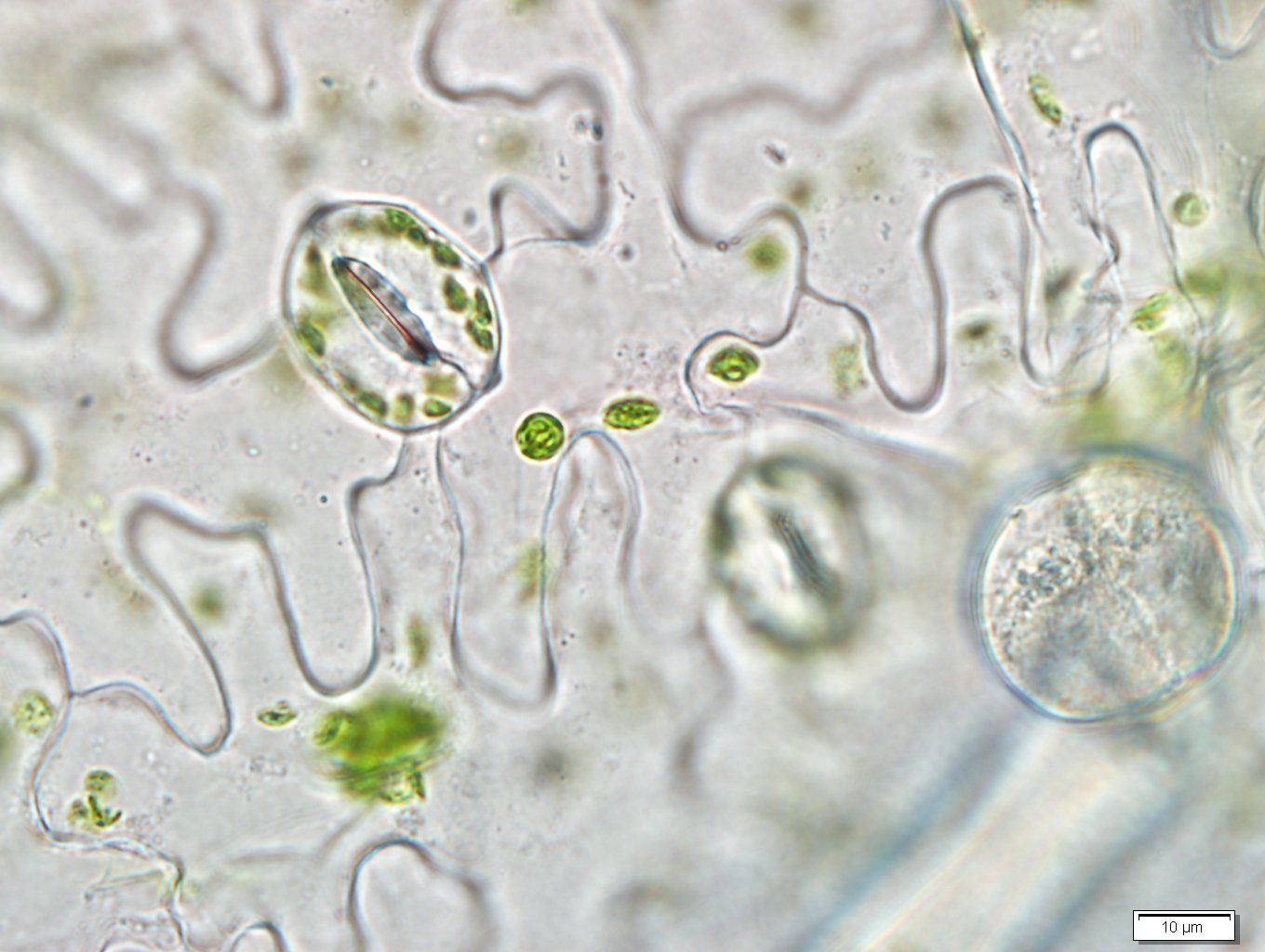

Supplement: S1 File — (ZIP) [file pone.0157026.s001.zip › FIG8/FIG8 OE3 NaCl.jpg]

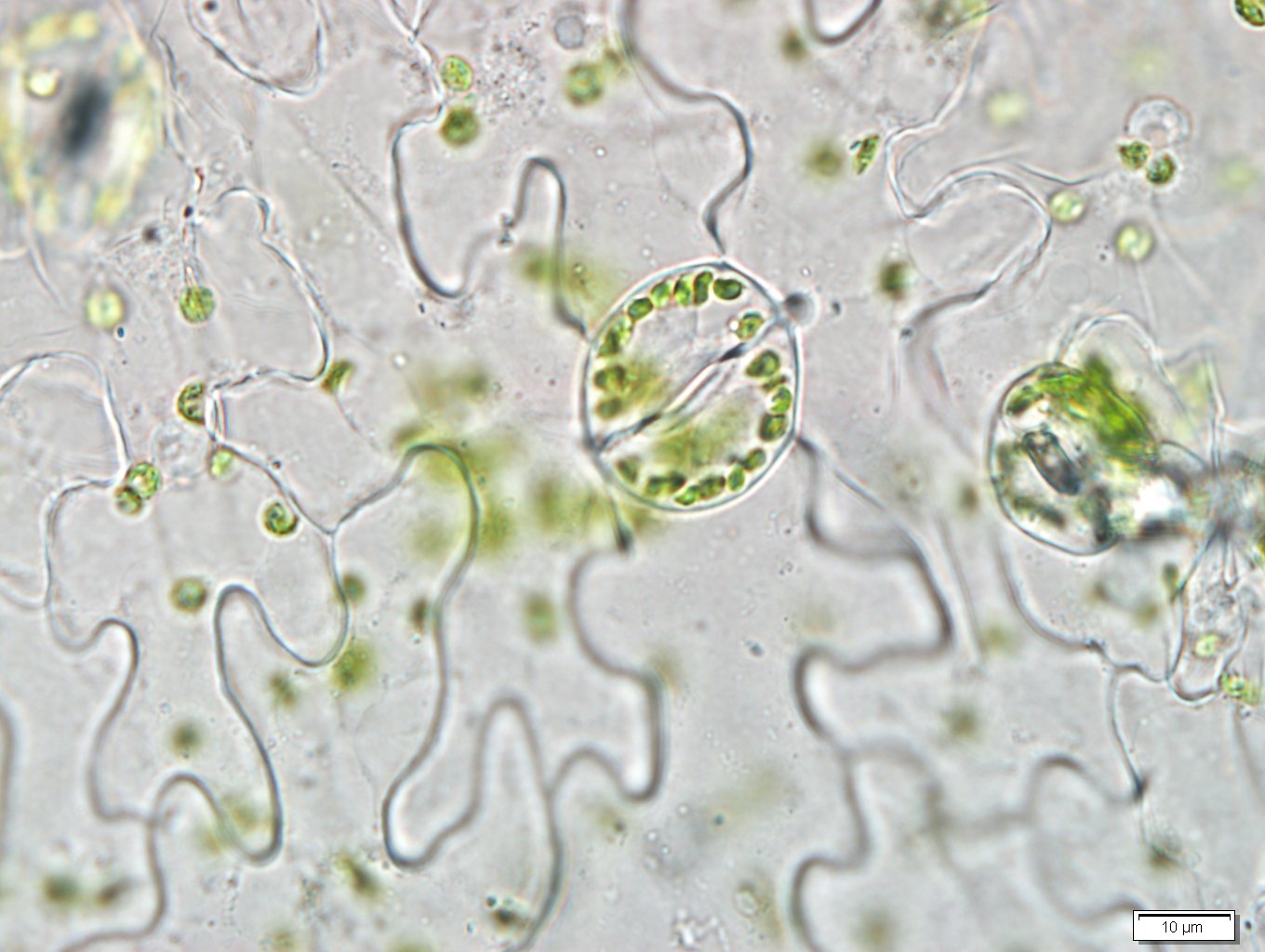

Supplement: S1 File — (ZIP) [file pone.0157026.s001.zip › FIG8/FIG8 OE3 PEG.jpg]

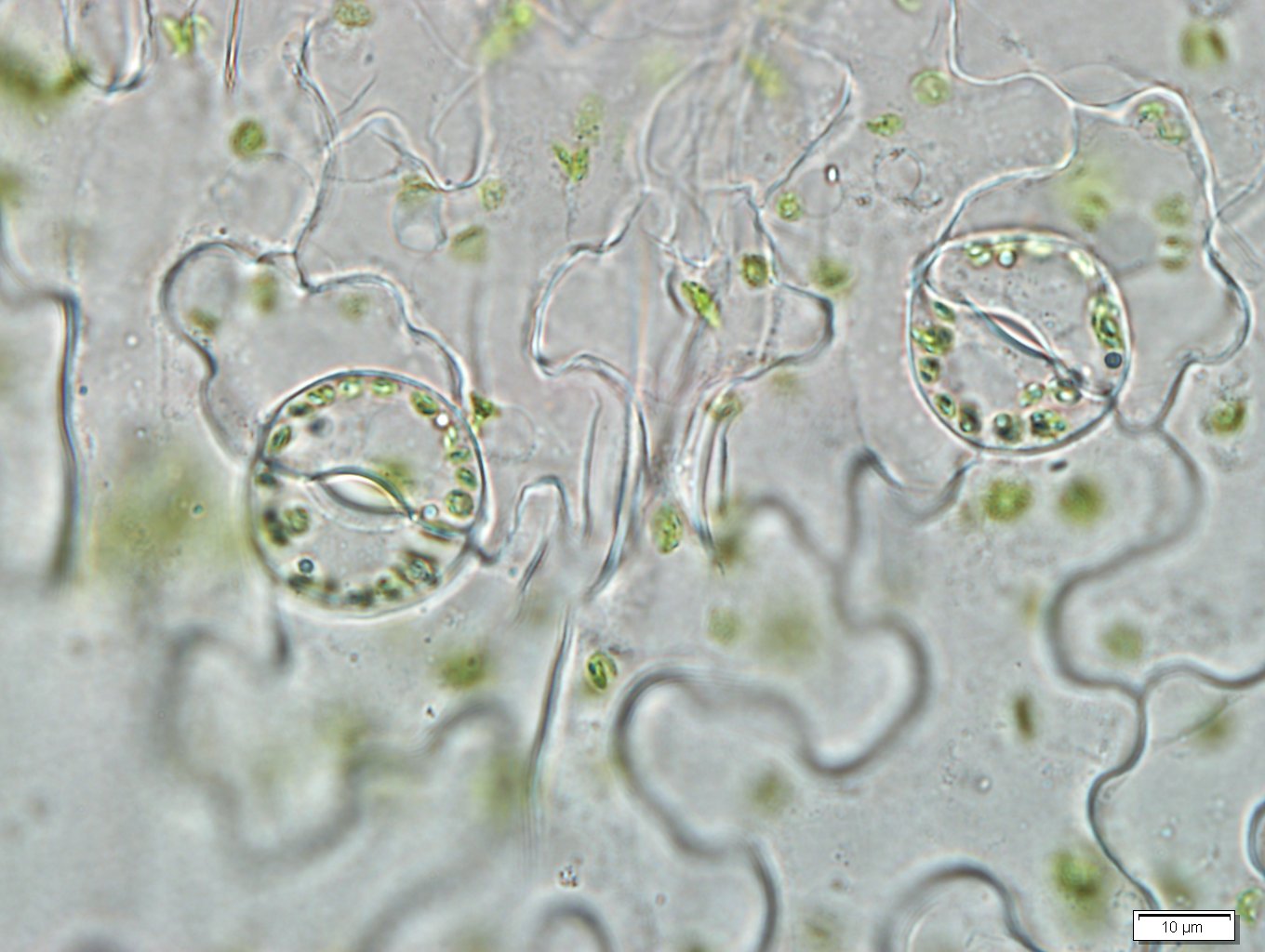

Supplement: S1 File — (ZIP) [file pone.0157026.s001.zip › FIG8/FIG8 OE3 control.jpg]

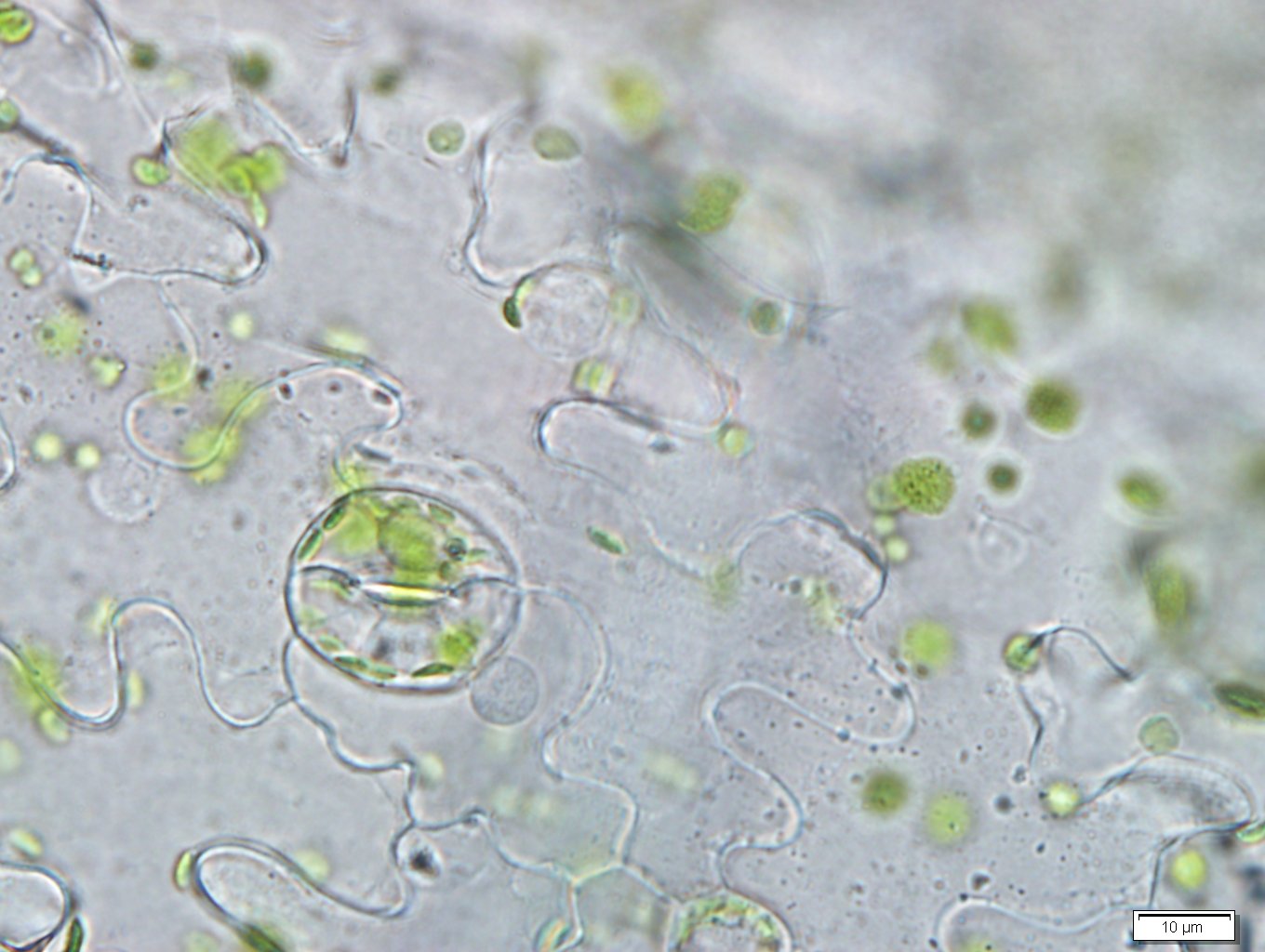

Supplement: S1 File — (ZIP) [file pone.0157026.s001.zip › FIG8/FIG8 WT NaCl.jpg]

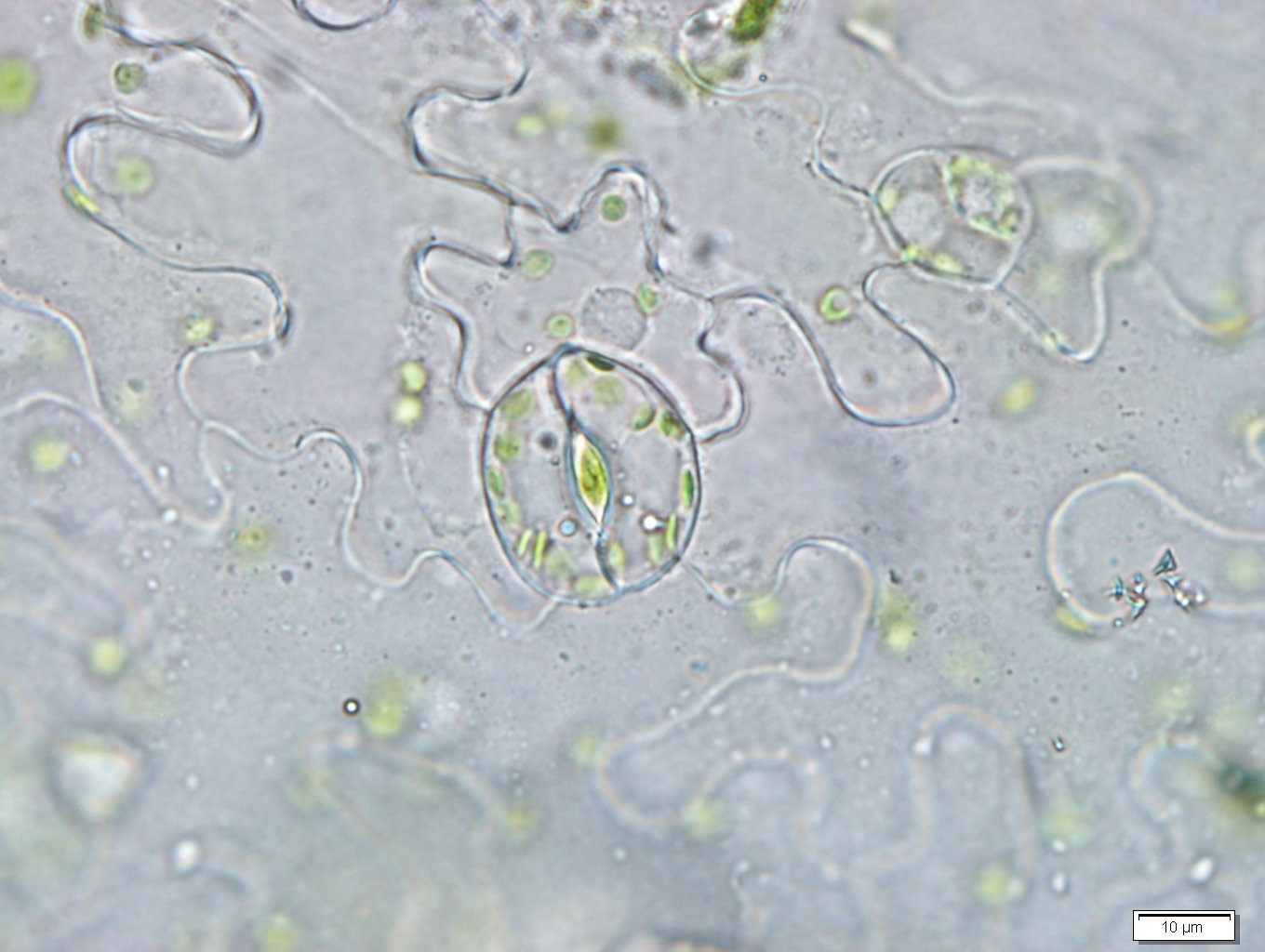

Supplement: S1 File — (ZIP) [file pone.0157026.s001.zip › FIG8/FIG8 WT PEG.jpg]

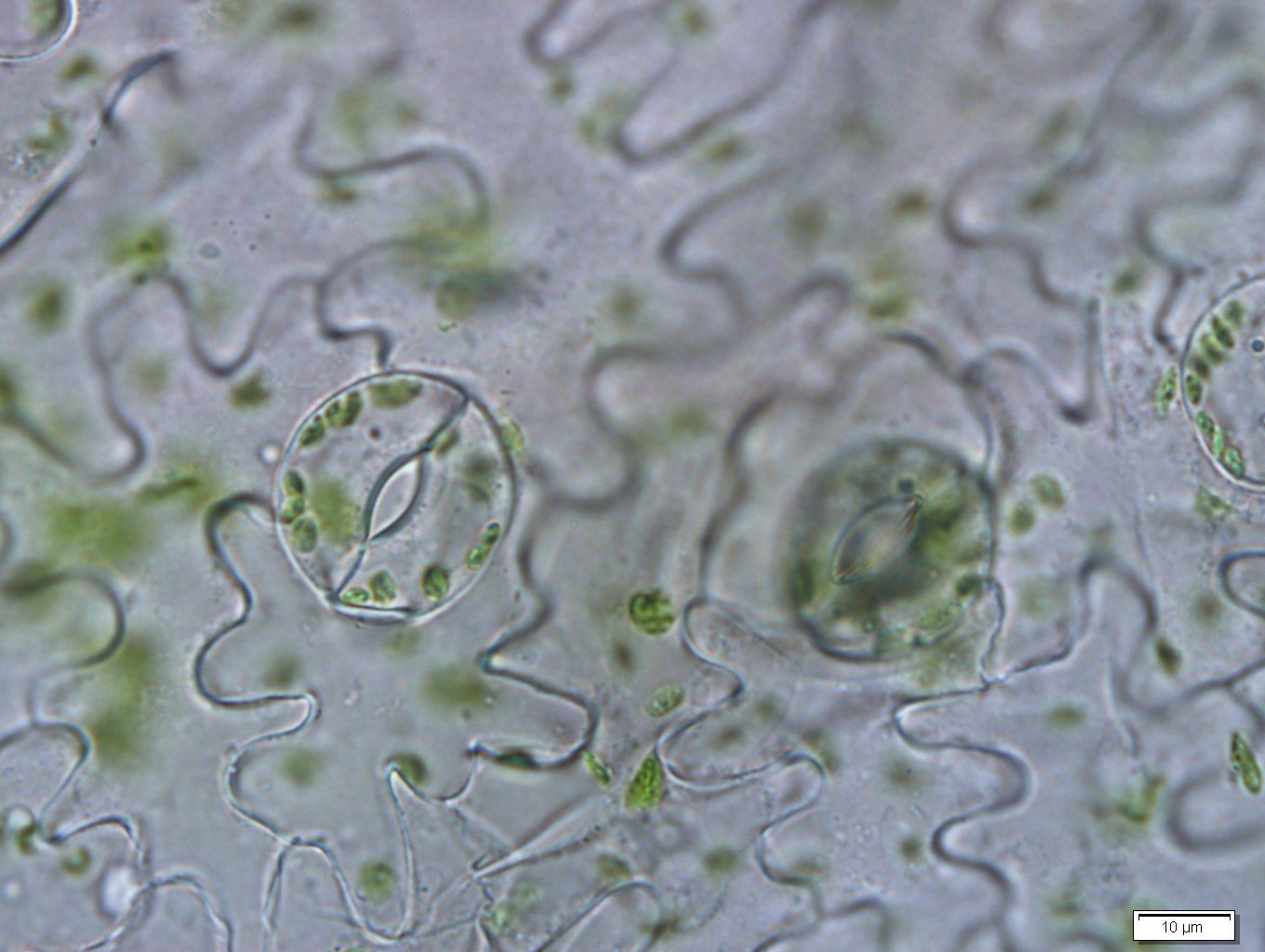

Supplement: S1 File — (ZIP) [file pone.0157026.s001.zip › FIG8/FIG8 WT control.jpg]
